# Phylogeographic structure, cryptic speciation and demographic history of the sharpbelly (*Hemiculter leucisculus*), a freshwater habitat generalist from southern China

**DOI:** 10.1186/s12862-017-1058-0

**Published:** 2017-09-12

**Authors:** Weitao Chen, Zaixuan Zhong, Wei Dai, Qi Fan, Shunping He

**Affiliations:** 1 The Key Laboratory of Aquatic Biodiversity and Conservation of Chinese Academy of Sciences, Institute of Hydrobiology, Chinese Academy of Sciences, Wuhan, Hubei 430072 China; 2 University of Chinese Academy of Sciences, Beijing, 100049 People’s Republic of China

**Keywords:** Phylogeographic structure, Cryptic speciation, *Hemiculter leucisculus*, Southern China, Multilocus

## Abstract

**Background:**

Species with broad distributions frequently divide into multiple genetic forms and may therefore be viewed as “cryptic species”. Here, we used the mitochondrial cytochrome *b* (Cytb) and 12 nuclear DNA loci to investigate phylogeographic structures of the sharpbelly (*Hemiculter leucisculus*) in rivers in southern China and explored how the geological and climatic factors have shaped the genetic diversity and evolutionary history of this species.

**Results:**

Our mitochondrial phylogenetic analysis identified three major lineages (lineages A, B, and C). Lineages B and C showed a relatively narrower geographic distribution, whereas lineage A was widely distributed in numerous drainages. Divergence dates suggested that *H. leucisculus* populations diverged between 1.61–2.38 Ma. Bayesian species delimitation analysis using 12 nuclear DNA loci indicated the three lineages probably represented three valid taxa. Isolation-with-migration (IM) analysis found substantial gene flow has occurred among the three lineages. Demographic analyses showed that lineages B and C have experienced rapid demographic expansion at 0.03 Ma and 0.08 Ma, respectively.

**Conclusions:**

*Hemiculter leucisculus* populations in drainages in southern China comprise three mtDNA lineages, and each of which may represent a separate species. Intense uplift of the Qinghai–Tibetan Plateau, evolution of Asian monsoons, changes in paleo-drainages, and poor dispersal ability may have driven the divergence of the three putative species. However, gene flow occurs among the three lineages. Climatic fluctuations have a prominent impact on the populations from the lineages B and C, but exerted little influence on the lineage A.

**Electronic supplementary material:**

The online version of this article (10.1186/s12862-017-1058-0) contains supplementary material, which is available to authorized users.

## Background

In recent years, numerous phylogeographical studies have shown that species with wide distribution ranges are frequently subdivided into genetically distinct groups [1–4]. The members of each group often cannot be discriminated by morphology and could represent hidden diversity. The detection and delimitation of such cases contribute to the understanding of speciation and its underlying processes; such studies increase our knowledge of the biodiversity of numerous taxa and are important guides for the conservation and management of biodiversity [5]. In some cases (e.g., *Haloptilus longicornis* [6] and *Microtus agrestis* [1]), these main genetic groups can be classified as “cryptic species” and the process generating such groups can be called “cryptic speciation”.

Recent improvements in molecular and analytical tools have aided in investigations of cryptic speciation. Multilocus sequence data involving newly developed theoretical models can be particularly helpful to generate species trees of closely related taxa [7]. Several methods have been developed to address the incongruence between gene trees and species trees due to incomplete lineage sorting [7–9].

Geologic changes and climatic instabilities are important factors that influence the evolutionary history of many taxa. Geologic changes have frequently shape the genetic structure of many species in East Asia [10–14], including fish species [13, 15]. For example, the uplift of the Qinghai–Tibetan Plateau [16, 17] has been reported to drive speciation and population diversification in a number of East Asian fish species [3, 18, 19]. Substantial changes in paleodrainage patterns in southwestern China due to the orogenesis of the Qinghai–Tibetan Plateau [20] also exerted a more direct influence on genetic divergence among fish populations [21, 22].

Climatic instabilities during the Quaternary Period have resulted in periodic expansions and contractions of the population sizes and distribution ranges of species [23, 24]. For example, in regions of Europe and North America, cyclical glacial fluctuations shaped the distributions of species and their genetic attributes [24, 25]. However, major glaciations did not occur in East Asia [26, 27], and a relatively mild Pleistocene climate prevailed in southern China [28, 29]. Therefore, climatic cycling may have exerted little effect on population demographies in southern China [30, 31]. Nevertheless, exceptions can be seen in some plants and animals [32–35].

Cyprinids represent one of the most diverse freshwater fish groups and are a major component of the primary freshwater ichthyofauna of Eurasia [36]. The wide distribution of cyprinids makes these taxa of particular interest for studying evolutionary history and testing biogeographic hypotheses. Cyprinids achieve their highest diversity in Asiatic waters and represent more than 50% of freshwater fish diversity in the major Asian rivers, inlcluding the Yangtze River [37] and Mekong River [38].

Species with broad ranges are frequently found to show diverse population structure and distinct evolutionary histories [3, 10, 11, 31, 39, 40]. This is the case for some cyprinids in East Asia rivers, e.g., *Rhynchocypris oxycephalus* [3], *Opsariichthys bidens* [41] and *Zacco platypus* [42]. The sharpbelly, *Hemiculter leucisculus* (Cyprinidae: Cultrinae), a small cyprinid fish, reaches a size up to 23.0 cm long, and is native to fresh and brackish water habitats with a pH of 7.0, a hardness of 15 dH, and a temperature of 18 to 22 °C [43]**.** This species has a wide distribution in the drainage basins of China, North Korea, South Korea, Japan, Mongolia and Russia (Additional file 2: Figure S1). In China, *H. leucisculus* widely occupies various freshwater habitats, such as rivers, lakes, reservoirs, and even pools [43, 44]. Habitat generalist and wide distribution ranges of the *H. leucisculus* populations make this species an ideal candidate for the study of phylogeographic structure and for the test of biogeographic hypotheses. In addition, with the increasing introduction of commercial fishes, some *H. leucisculus* populations have been imprudently introduced into many nonnative distributions [44].

Understanding the evolutionary history and speciation process of the sharpbelly provides an important case of the influence of biogeographic process on East Asia fish species and is important to manage this species in the future. In the current study, therefore, we use both mitochondrial DNA (mtDNA) and multiple nuclear DNA (nDNA) markers to investigate the phylogeographical structure and evolutionary history of the *H. leucisculus* populations in the rivers in southern China. In addition, we attempt to discuss the implications of our phylogeographic results in identifying cryptic diversity in *H. leucisculus*. Lastly, we evaluate the possible effects of geologic changes and climatic instabilities on the evolutionary history of *H. leucisculus* populations.

## Methods

### Ethics statement

All experimental protocols were approved by the Ethics Committee of the Institute of Hydrobiology, Chinese Academy of Sciences. The policies were enacted according to Chinese Association for Laboratory Animal Sciences, and coordinated with the Institutional Animal Care and Use Committee (IACUC) protocols (http://iacuc.usc.edu/).

### Sampling

**Fig. 1:**
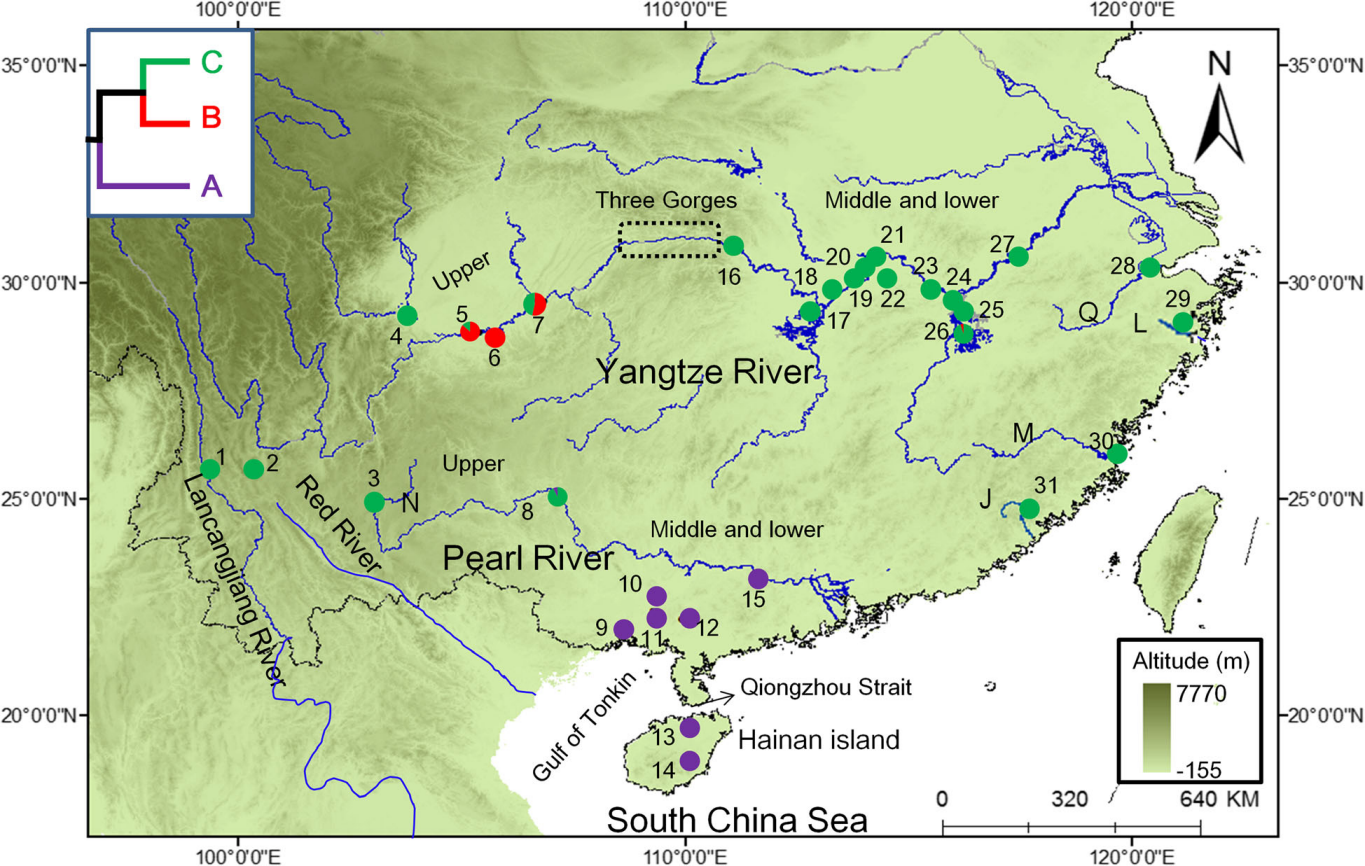
Map of sampling locations of *Hemiculter leucisculus*. Localities are detailed in Additional file 1: Table S1 and populations are presented as pie charts with slice sizes proportional to the frequency of the three lineages (see Fig. 2), as identified by phylogenetic analysis. Colors correspond to the lineages (purple, lineage A; red, lineage B; green, lineage C). Localities 15 and 27 are sample sites without exact orientation. H, Hainan island; J, Jiulongjiang River; L, Lingjiang River; M, Minjiang River; N, Nanpanjiang River; Q, Qiangtangjiang River

A total of 381 specimens of *H. leucisculus* from 22 localities were collected from the Lancangjiang River, Pearl River and Yangtze River basins (Additional file 1: Table S1, Fig. 1). A small piece of white muscle tissue or fin was dissected from the right side of each specimen. All tissue for genomic DNA extraction was preserved in 95% ethanol. Voucher specimens were deposited in the collection of the Freshwater Fish Museum of the Institute of Hydrobiology, Chinese Academy of Sciences.

The total genomic DNA was extracted from muscle tissue or fin tissue using standard salt extraction [45]. The partial mitochondrial cytochrome *b* gene (Cytb) was amplified for all individuals using the universal primers L14724 and H15915 [22]. We sequenced 98 individuals de novo and retrieved 283 sequences from a previous study [46]. Nine published sequences were included in our analyses. Among the published sequences, six sequences were sampled from six small and coastal river basins, and the remaining sequences were sampled from the Pearl River and Yangtze River basins (Additional file 1: Table S1). A total of 390 Cytb sequences from 31 localities covering most of drainages in southern China were analysed (Additional file 1: Table S1). We sequenced up to 12 independent nuclear loci from a subset of individuals which covered the range of Cytb phylogenetic tree. These loci correspond to the protein-coding genes early growth response 3 (EGR3), ectodermal-neural cortex 1 (ENC1), glycosyltransferase (Glyt), myosin heavy chain 6 (myh6), pleiomorphic adenoma gene-like 2 (plagl2), peptide transport (Ptr), recombination activating gene 2 (RAG2), Rhodopsin, ryanodine receptor 3 (RYR3), SH3 and PX domain containing 3 (SH3PX3), sterol regulatory element-binding protein 2 (sreb2) and zic family member 1 (zic1) (Additional file 2: Table S2). Primer information is listed in Additional file 2: Table S2. The sample size of 12 nuclear genes ranged from 26 to 55 individuals, which derived from 20 populations (Additional file 1: Table S1; Additional file 2: Table S3). These markers were amplified and sequenced following a previous protocol [47–49]. The amplification of genomic DNA was conducted in a 30-μL reaction with an initial denaturation period of 3–5 min at 94 °C followed by 30–35 cycles of 94 °C for 0.5–1 min, primer-specific annealing temperature of 53–64 °C (Additional file 2: Table S2) for 0.5–1 min, 72 °C for 1–1.5 min and a single final extension at 72 °C for 5–10 min. The amplified fragments were purified using 1.0% low-melting agarose gel electrophoresis and sequenced with the identical primer pair using an ABI PRISM 3700 (Applied Biosystems) automatic DNA sequencer.

### Sequence analyses

The nucleotide sequences were initially edited using the DNASTAR multiple package (DNASTAR Inc., Madison, WI, USA), aligned using MUSCLE [50] and optimized by eye using MEGA version 6.0 [51]. For the nuclear genes, the polymorphic positions for individual sequences from nuclear loci were carefully inspected to ensure correct and consistent identification of double peaks in heterozygotes. Nuclear gene sequences containing more than one ambiguous site were resolved using PHASE 2.1.1 [52, 53], for which input files were prepared using SEQPHASE [54]. Three runs were performed for each locus with default settings. Recombination tests for detecting the longest non-recombining region for each locus were conducted using IMGC [55].

### Phylogenetic analyses

Phylogenetic analyses of *H. leucisculus* of the Cytb data were reconstructed using Bayesian inference (BI) and maximum-likelihood (ML) methods. *Hemiculter bleekeri* (GenBank no: KF029693) and *Culter alburnus* (GenBank no: KM044500) downloaded from GenBank, were used as the outgroup. Nucleotide substitution models (GTR + I + G) were selected using the Akaike information criterion in MRMODELTEST version 2.3 [56]. For BI analyses, three independent runs and four Markov chains (three heated chains and a single cold chain) using the best-fit models were performed in MRBAYES v 3.1.2 [57], starting from a random tree. Each run was conducted for 20 million generations and sampled every 1000 generations, with the first 25% discarded as burn-in. We checked for stationarity by ensuring that the potential scale reduction factor equaled 1 and that the average standard deviation of split frequencies between independent runs approached 0. We examined the MCMC samples in TRACER 1.5 [58] to ensure that all chains were sampled from the same target distribution. ML analyses were implemented in RAXML-VI-HPC [59] using a GTR + I + G model. Nodal support values were estimated from 1000 nonparametric bootstrap replicates. Nodes with 95% Bayesian posterior probabilities in the BI analysis [60] and nodes with 75% bootstrap support in the ML analysis were considered strongly supported. We also used NETWORK 4.6 [61] to construct a median-joining network for Cytb.

In addition, BI and ML analyses were employed for the nDNA loci using the identical parameter settings as well. The optimal nucleotide substitution model of each nDNA locus (Additional file 2: Table S4) was chosen using the Akaike information criterion in MRMODELTEST.

### Bayesian species delimitation

To examine whether the three lineages inferred from Cytb trees represent different species, we used Bayesian Phylogenetics and Phylogeography (BPP) v3.0 [7, 62] with 12 nuclear genes. This method allowed for the absence of monophyly for DNA haplotypes and incomplete lineage sorting [7, 63]. The model of species delimitation rjMCMC algorithm0 and finetune(e) was used in this analyses. Because the prior distributions of the ancestral population size (ɵ) and root age (τ_0_) could have affected the posterior probabilities of the models [63], three different combinations of ɵ and τ_0_ were implemented in BPP: (ɵ = (1, 10) τ = (1, 10); ɵ = (2, 2000) τ = (2, 2000); and ɵ = (1, 10) τ = (2, 2000). We ran the rjMCMC analyses for 1,000,000 generations, sampled every five generations, and discarded samples from the first 50,000 generations as burn-in. Each analysis was repeated at least twice to confirm consistency between runs. Topology based on Cytb was used as a guide tree.

### Divergence time estimation

Divergence times among the major lineages of *H. leucisculus* were incorporated in the program BEAST 1.6.1 [64]. For molecular time estimation, the molecular clock was assessed by relative-rate tests using PHYLTST [65]. Lacking of fossil evidence, divergence times were estimated according to the strict-clock model. Based on the mitochondrial Cytb data, a range of substitution rate of 1.00–2.00% per million years (Myr) is often adopted, as these rates have been widely employed for mitochondrial Cytb gene analysis in cyprinid fish [66–68]. Thus, we employed the average value (1.50% per Myr) of the above rate range for Cytb in our study. Two independent runs were run, and the output .log and .tre files were combined using LogCombiner (BEAST package). A Yule process was implemented for the tree prior model, and the GTR + I + G model of nucleotide substitution was implemented. Each run was conducted for 50 million generations and sampled every 1000 iterations. The trees were summarized with TreeAnnotator v.1.6.1 (BEAST package) into the maximum clade credibility tree and the first 25% of sampled trees were treated as burn-in. Burn-in and convergence of the chains were determined with TRACER 1.5. ESS were used to determine the Bayesian statistical significance of each parameter.

### Gene flow

Potential gene flow among major lineages was estimated using the isolation-with-migration (IM) model in the IMa2 program [69]. We used Cytb and the longest non-recombining regions of the 12 nuclear loci for IM analyses. The Cytb tree was used as the guide tree. The method estimates the density functions and posterior-probability densities of the IM model parameters using a Markov chain (MCMC) method [70]. The functions of the model parameters were first estimated in M-mode with two million generations, and the first 10% were discarded as burn-in. The MCMC run was repeated three times to confirm convergence. Using these functions, the marginal posterior distribution and the maximum-likelihood estimates of the demographic parameters were estimated in the L-mode. The HKY model of the DNA substitution was employed for all markers, and 40 heated metropolis-coupled Markov chains were employed to ensure convergence. ESS were used to determine whether the convergence was satisfactory.

### Genetic diversity and population structure based on Cytb sequences

The number of haplotypes (n), private haplotypes (ph), haplotype diversity (*h*) and nucleotide diversity (π) of each population and lineage were calculated using DnaSP v5 [71]. In addition, pairwise divergences between and within the lineages were estimated by Kimura’s (1980) two-parameter [72] as implemented in MEGA version 6.0.

To investigate population structure, an analysis of molecular variance (AMOVA) [73] was performed in ARLEQUIN 3.5 [74] using Jukes and Cantor distances [75]. Populations were grouped into three groups (Lineages A, B, and C) suggested by the mtDNA phylogenetic analysis and grouped into nine groups based on drainage system. Pairwise genetic differentiation (ɸ_ST_) was calculated using Jukes and Cantor distances between populations having more than five samples (17 populations fulfilled this condition) in ARLEQUIN 3.5. A Mantel test was used to investigate isolation by distance using genetic differentiation (ɸ_ST_/(1-ɸ_ST_)) and the geographical distance (in kilometers) measured as the straight-line distance, in ARLEQUIN 3.5. Geographic distances were estimated using GOOGLE EARTH v.4.3. All calculations implemented in ARLEQUIN 3.5 used 1000 permutations to assess significance.

### Demographic estimation

Sample size allowing observed lineages was screened for historical demographic events in each deme. Selective neutrality test of Fu’s *Fs* statistics [76] based on Cytb was conducted for each lineage using ARLEQUIN 3.5 to find evidence of recent expansion. Significantly negative values are expected in populations that have undergone recent population expansion [76, 77]. We assessed significance by generating null distributions from 1000 permutations. To understand how population sizes changed though time, we examined the historical demographics of all lineages using coalescent-based extended Bayesian skyline plots (EBSPs). EBSPs were constructed using Cytb and three randomly selected nuDNA loci (EGR, Ptr and RAG2) in BEAST 1.6.1 to assess the variation in effective population sizes through time. The best model of nucleotide substitution was selected for each lineage and each loci (Additional file 2: Table S5), and time was scaled using a substitution rate of 1.50% substitutions/million years for Cytb data. The evolutionary rates for three nuDNA loci were estimated as a function of the Cytb evolutionary rate. A strict clock model was set as prior, 100 million generations were run for the three lineages. Each EBSP was sampled every 1000 iterations, with 10% of the initial samples discarded as burn-in. Stationarity was assessed by analyzing the effective sizes of all parameters in TRACER 1.5.

## Results

### Sequence information

A 1040-bp segment of Cytb was obtained from 390 individuals of *H. leucisculus* including 98 novel sequences, 283 sequences from the study by Fan & He (2014) [46] and 9 sequences from NCBI. The 390 sequences contained 213 variable sites and 150 parsimony-informative sites. A total of 171 unique haplotypes were defined from ingroup sequences. In addition, our nuclear dataset consisted of 12 independent loci with lengths ranging from 676 bp (Ptr) to 1227 bp (RAG2) (Additional file 2: Table S3). The number of individuals, the length of each nDNA locus, the variable sites and the informative parsimony sites are showed in Additional file 2: Table S3. We translated all sequences into amino acids, and no stop codon was detected. All novel sequences analyzed in this study have been deposited in GenBank under Accession nos KY292565–KY293216 (Additional file 1: Table S1).

### Phylogenetic analyses of Cytb and nDNA genes

**Fig. 2:**
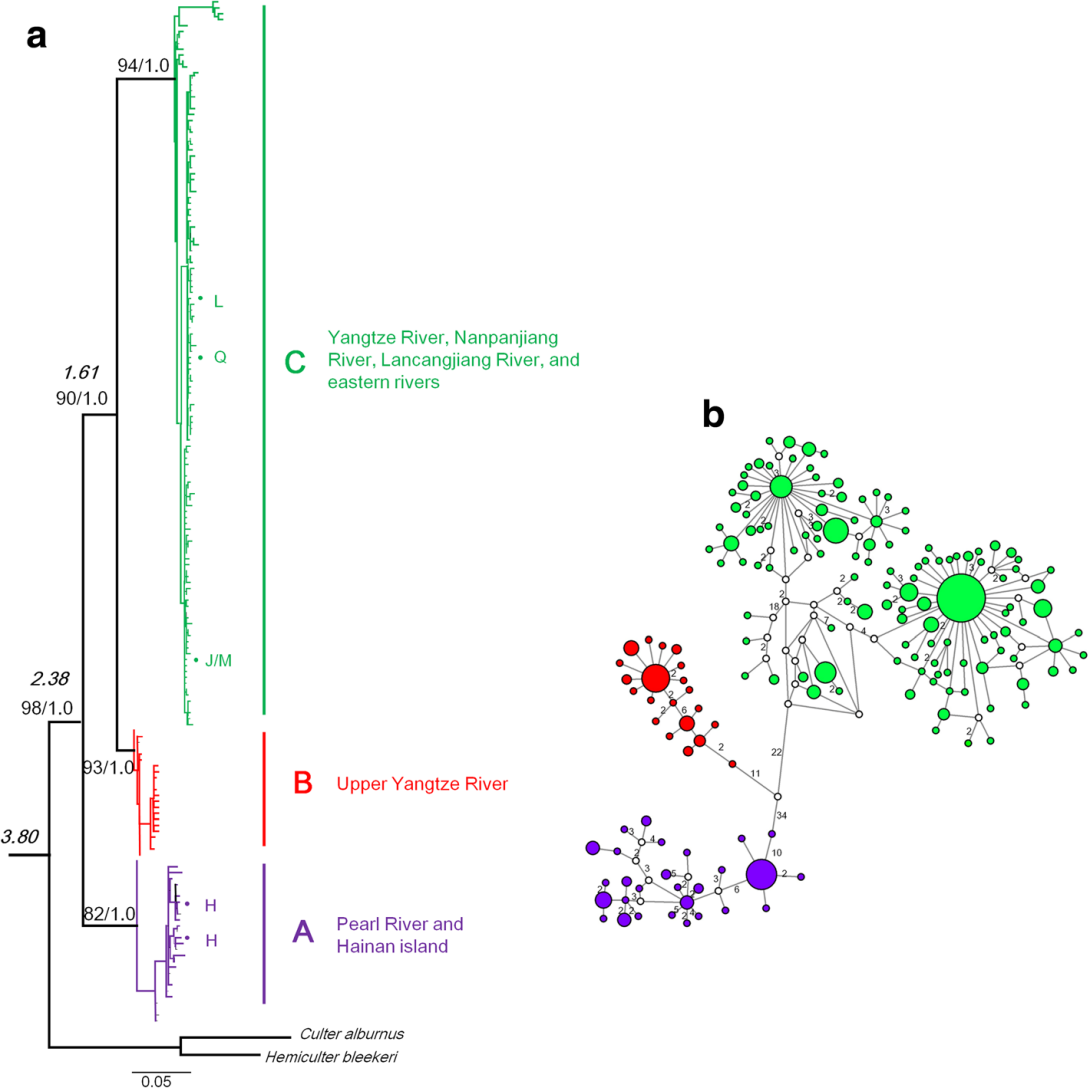
**a** Phylogenetic tree based on Bayesian inference showing the relationships among *Hemiculter leucisculus* populations for the cytochrome *b* gene (Cytb). Values on branches indicate bootstrap proportions from a maximum likelihood analysis and Bayesian posterior probabilities. Numbers in bold and italic indicate divergence time estimates for *Hemiculter leucisculus*. H, Hainan island; J, Jiulongjiang River; L, Lingjiang River; M, Minjiang River; N, Nanpanjiang; Q, Qiangtangjiang River. **b** Median-joining network of Cytb sequences. Each colored circle represents different lineages, scaled according to its frequency in the entire sample. An empty circle indicates missing intermediate steps between observed haplotypes, and the numbers indicate mutation steps between haploptypes

The ML and BI trees based on Cytb produced similar gene tree topologies and congruently revealed three well-supported monophyletic lineages (only BI tree shown, Fig. 2a). Lineage A, the sister group of the other two lineages, consisted of specimens from the Pearl River basin and Hainan Province (Fig. 1). Lineage B, the sister group of lineage C, mainly occurred in a small area (Localities 5–7) in the upper branch of the Yangtze River (Fig. 1). Lineage C included samples from most rivers in southern China, e.g., Yangtze River, the Nanpanjiang River, the Lancangjiang River (upper Mekong), and several eastern rivers (Fig. 1). Twelve individuals sampled from Locality 8, which is within the Pearl River basin, were also grouped into this lineage. In addition, lineages A and B displayed a high degree of geographic structure, whereas lineage C revealed a poor relationship with geographic distribution (Fig. 1). The network analyses for Cytb (Fig. 2b) showed similar patterns to the gene tree (Fig. 2a).

**Fig. 3:**
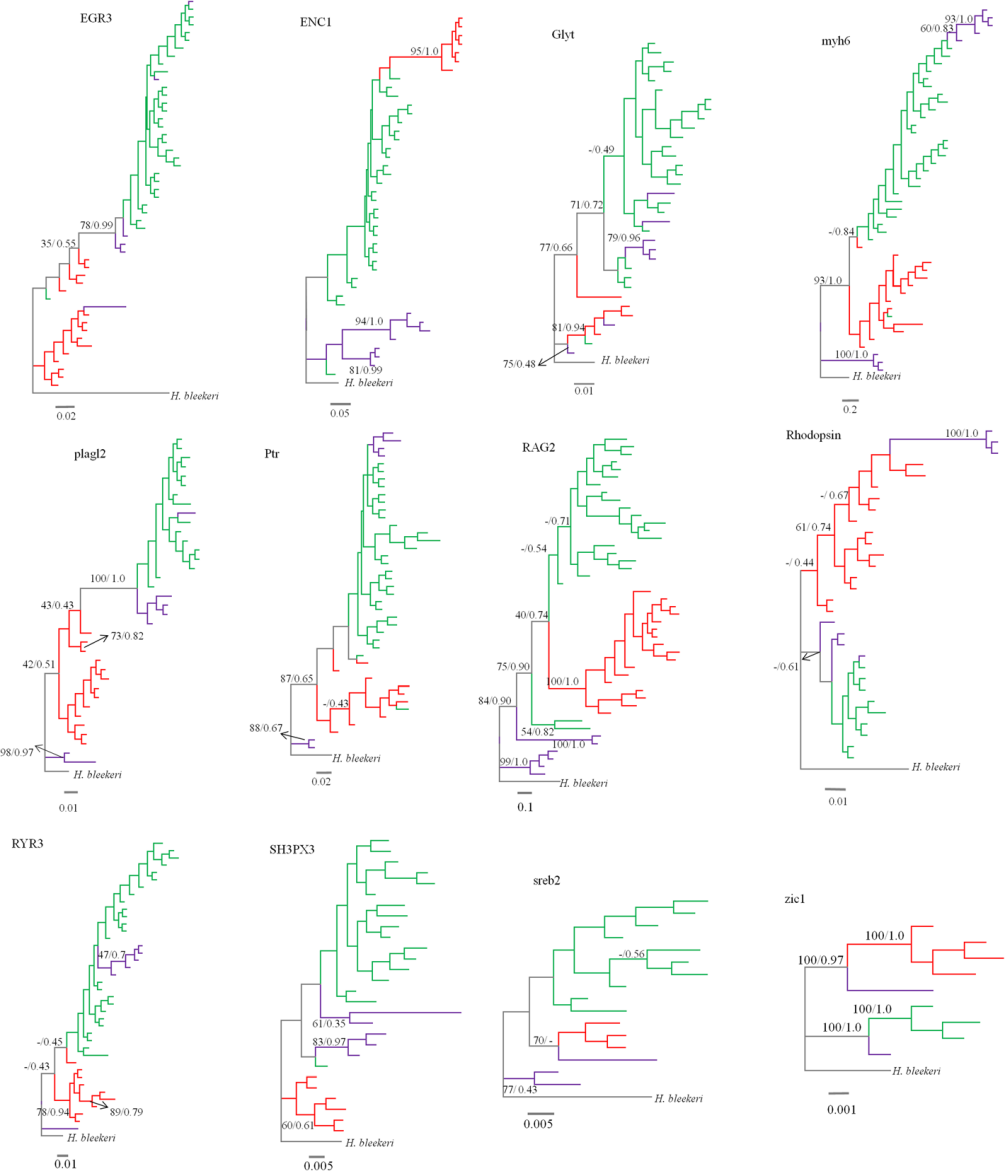
Phylogenetic trees based on Bayesian inference showing the relationships among the lineages for the 12 nuclear genes. Values on branches indicate bootstrap proportions from a maximum likelihood analysis and Bayesian posterior probabilities. Colors correspond to the lineages (purple, lineage A; red, lineage B; green, lineage C)

The nDNA phylogenetic trees showed that members of the three mtDNA lineages can be distinguished with three nDNA genes (i.e. ENC1, RAG2 and zic1), although the support is not very strong in some nodes (Fig. 3). The three lineages did not group into reciprocally independent clusters in the phylogenetic trees based on nine other nDNA genes (Fig. 3). Furthermore, the support value or Bayesian posterior probability of the nine nDNA trees was low in most nodes (Fig. 3).

### Bayesian species delimitation

**Fig. 4:**
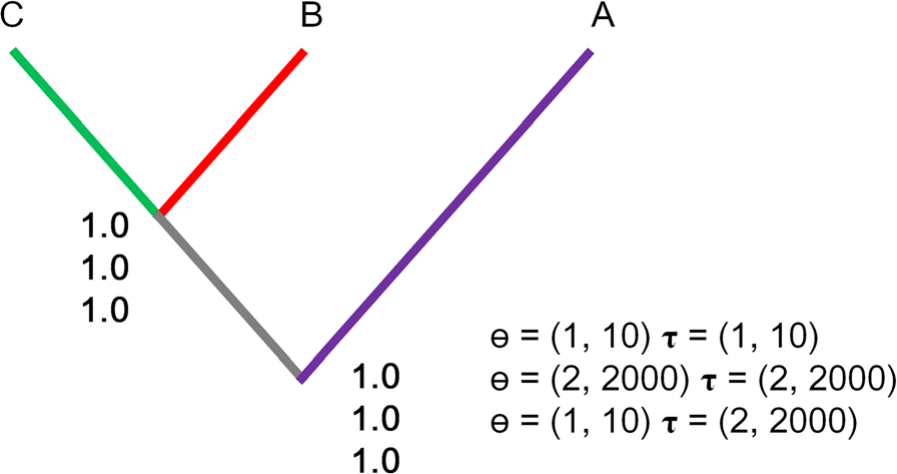
Bayesian species delimitation results for *Hemiculter leucisculus* assuming three species. The speciation probabilities are provided for each node under each combination of priors for ɵ and τ_0_. Colors correspond to the lineages (purple, lineage A; red, lineage B; green, lineage C)

The nuclear data strongly supported that the three lineages defined by the Cytb data represent three different species with perfect statistical support (PP = 1.00; Fig. 4). Different sets of prior distributions for ɵ and τ_0_ did showed consistent results.

### Divergence dating

The null hypothesis of clock-like evolution was not rejected at the 5% level. Therefore, we estimated the divergence times for the monophyletic Cytb lineages identified in the phylogenetic analyses (lineages A, B, and C as labeled in Fig. 2) using a strict molecular clock. The dating analyses (Fig. 2a) suggested that the most recent common ancestor of *H. leucisculus* diverged at 3.80 Ma (95% highest posterior density (HPD) = 2.92–4.65 Ma). Lineage A diverged at 2.38 Ma (95% HPD, 1.82–2.93 Ma), and lineages B and C split at 1.61 Ma (95% HPD, 1.19–1.98 Ma).

### Gene flow among the three lineages

**Fig. 5:**
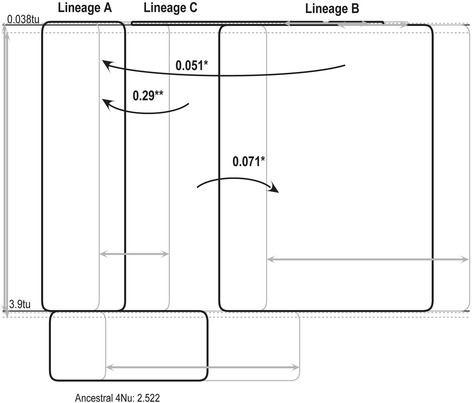
Gene flow analyses for the three lineages of the *Hemiculter leucisculus* popualtions. The arrows represent migration directions from the source population to the receiving population; the numbers next to arrows are 2NM values; grey arrows and boxes were marginal distribution values in demographic units. tu, time since ancestral population splitting in mutational units. Only statistically significant cases of gene flow are presented. ^*^ *P* < 0.05 and ^**^ *P* < 0.01

We detected three statistically significant (*P* < 0.05) and unidirectional migration events among the three lineages (Fig. 5). The three migration events were from lineages C to B with a population migration rate 2NM = 0.071, from lineages C to A with 2NM = 0.29, and from lineages B to A with 2NM = 0.051.

### Genetic diversity and population structure

Overall *h* was high (0.9737 ± 0.0043), with the highest within-lineage *h* occurring in lineage C (*h* = 0.9573 ± 0.0082), and was similar in lineages A (*h* = 0.868 ± 0.0400) and B (*h* = 0.891 ± 0.0320) (Table 1). Overall π was 0.0290 ± 0.0013, with the highest within-lineage π in lineage A and lowest in lineage B (Table 1). The number of haplotypes (n), private haplotypes (ph), *h*, and π varied among populations (Additional file 2: Table S6). A total of 134 private haplotypes were defined in all populations. The uncorrected K2P distance within each lineage is listed in Table 1. The largest distance within lineages was found in the lineage A (0.82%), the distance was intermediate in the lineage C (0.73%), and smallest in the lineage B (0.45%). The interlineage genetic distances (Additional file 2: Table S7) ranged from 4.39% (between lineages A and B) to 6.87% (between lineages A and C).

**Table 1:** Genetic diversity and neutrality test statistics for each lineage based on Cytb

	n/N	*h*	π	*Fu’s*	K2P
A	28/65	0.891 ± 0.0320	0.0081 ± 0.0004	−5.36	0.82%
B	21/50	0.868 ± 0.0400	0.0044 ± 0.0005	−6.72^*^	0.45%
C	122/275	0.957 ± 0.0082	0.0072 ± 0.0003	−24.40^***^	0.73%

N, individual numbers; n, haplotype numbers; *h*, haplotype diversity; π, nucleotide diversity; K2P, Kimura’s two-parameter (K2P) distance within each lineage. ^*^ *P* < 0.05, ^***^ *P* < 0.001

Results from the two-level hierarchical AMOVA suggested that *H. leucisculus* was highly structured geographically: 72.88% of the genetic variation was attributed to differentiation among populations, and this was highly significant (*P* < 0.001) (Table 2). Furthermore, the AMOVA based on the three lineages revealed that most of the variation (89.37%, *P* < 0.001) occurred among groups (Table 2). Lastly, the AMOVA based on drainage systems accounted for 59.21% of the overall variation among groups and accounted for 22.32% among populations within groups (Table 2). One hundred and one out of 136 pairwise ɸ_ST_ values were statistically significant ranging from 0.048 to 0.942 (*P* < 0.001 to *P* = 0.043). A significant correlation between the pairwise genetic differentiations (ɸ_ST_/(1-ɸ_ST_)) and the geographical distance was found over the entire dataset (Mantel test; *r* = 0.27, *P* = 0.015), indicative of isolation by distance (IBD).

**Table 2:** Results of an analysis of molecular variance (AMOVA) for three grouping options of *Hemiculter leucisculus* populations estimated using ɸ–statistics based on Cytb

Source of variation	Percentage of variation	ɸ_CT_	P
Grouped by clades			
Among clades	89.37	0.89	< 0.001
Within clades	10.63	0.11	
Grouped by drainages			
Among drainages	59.21	0.59	< 0.001
Among populations, within drainage	22.32	0.22	< 0.001
Within populations	18.47	0.18	< 0.001
Grouped by sample sites			
Among populations	72.88	0.73	< 0.001
Within populations	27.12	0.27	

### Demographic history

**Fig. 6:**
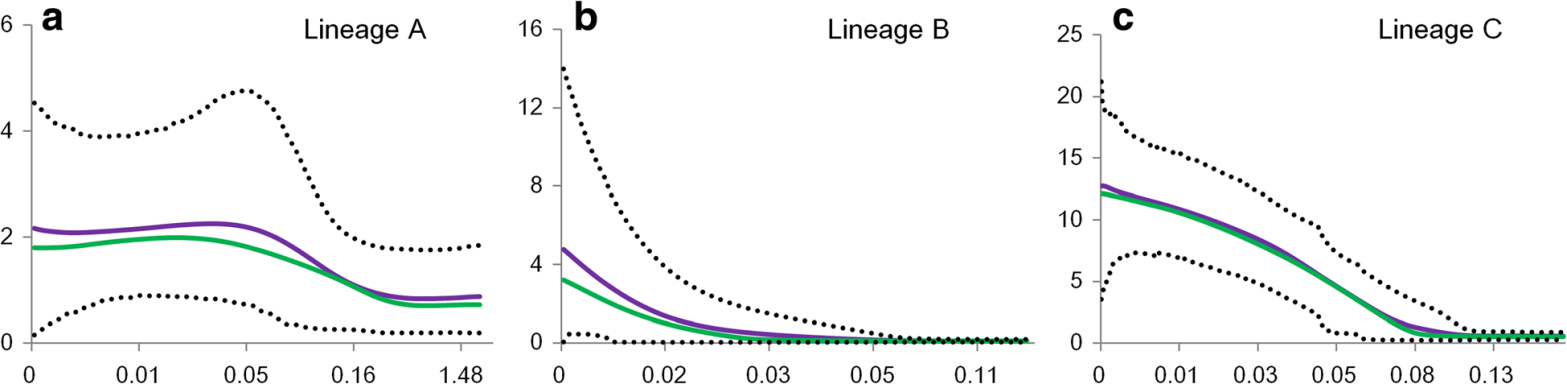
Historical demographic analysis for each lineage of *Hemiculter leucisculus* as inferred by extended Bayesian skyline plots (EBSP) analysis. *x*-axes correspond to time (Ma) and *y*-axes correspond to Neτ, the product of effective population size and generation length in years. Green and purple lines represent median and mean values for the log_10_ of the population size (*N*_e_*τ) (τ, generation time), respectively. Dashed lines mark the 95% highest probability density (HPD) intervals in all panels. **a**-**c** Extended Bayesian skyline plots for lineages A, B, and C, respectively

A neutrality test conducted on the Cytb suggested that lineages B and C experienced a demographic expansion, as indicated by significant negative values of Fu’s *F*_*S*_statistics (Table 1). Similar results were demonstrated by the EBSP analyses (Fig. 6). EBSPs suggested that demographic expansion occurred approximately 0.025 Ma for lineage B and approximately 0.08 Ma for lineage C. Neither Fu’s *F*_*S*_ nor EBSP detected significant expansion for lineage A.

## Discussion

### Distribution patterns and three lineages

In this study, we clarify the phylogeographical structure and hidden diversity of *H. leucisculus* in the drainages of southern China in detail. We confirmed the existence of three highly divergent mtDNA lineages (A, B, and C) based on Cytb sequences. Lineages A and B display a highly geographical structure. Members of lineage A originate from the middle and lower Pearl River drainage and Hainan Island, and most representatives of lineage B are limited to a small area (Localities 5–7) in the Upper Yangtze River. Regarding lineage A, the populations from middle and lower Pearl River drainages and Hainan Island were unexpectedly found to show a close relationship, though they are separated by the Qiongzhou Strait. This may be a result of the fact that the Gulf of Tonkin and the Qiongzhou Strait were once part of the coastal plain of the Asian continent during the Pleistocene glaciations [78, 79]. Populations from lineage B are restricted to the Upper Yangtze River, suggesting that they originated and developed from these regions.

In contrast, lineage C is poorly correlated with its geographic distribution and is extensively distributed in many isolated drainage basins, including the Lancangjiang River (Localities 1 and 2), the Upper Pearl River (Localities 3 and 8), the Yangtze River, the Minjiang River, the Jiulongjiang River, the Lingjiang River and the Qiangtangjiang River (Fig. 1). The close relationship of *H. leucisculus* populations between the Yangtze River basin and the four coastal rivers in southeastern China (i.e., the Jiulongjiang River, the Lingjiang River, the Minjiang River, and the Qiangtangjiang River) may due to their spatial proximity. The spatial proximity among these rivers enables *H. leucisculus* populations from the Yangtze River basin to expand to nearby rivers easily due to floods in summer. The hypothesis of active river capture in southwestern China [80] may explain the close relationship of populations between the Yangtze River and Upper Pearl River (Localities 3 and 8). Active river capture and reverse events may have caused the Yangtze River and Upper Pearl River to connect in some regions. However, no Cytb haplotype shared between Nanpanjiang River populations (Locality 3) and Yangtze River populations (Additional file 2: Table S6) indicated that there may have been rare gene exchange between the two popuations, at least in mtDNA level. Lower genetic diversity and high levels of Cytb haplotype sharing with the Yangtze River populations (Additional file 2: Table S6) indicated that the Lancangjiang River populations (Localities 1 and 2) likely originated from the Yangtze River populations due to imprudent introduction by human in company with the large-scale introduction of economically valuable fish half a century ago [44, 81, 82].

In addition to the three strongly supported mtDNA lineages, a high level of mtDNA genetic difference observed between the lineages (4.39-6.87%) supported a deep divergence among *H. leucisculus* populations (Additional file 2: Table S7). This finding is common in small freshwater fish species in China, e.g., *Zacco platypus* [42, 83], *Opsariichthys bidens* [41], *Rhynchocypris oxycephalus* [3] and *Garra orientalis* [79].

We speculate that *H. leucisculus* originated in the drainages of southern China at 3.80 Ma (95% HPD, 2.92–4.65 Ma), suggesting that this species originated near the beginning of the Late Pliocene [84, 85]. This period is broadly consistent with the intense uplift of the Qinghai–Tibetan Plateau and evolution of Asian monsoons during 3.6–2.6 Ma [86, 87]. The divergence time estimation between lineage A and lineages B and C is at ~2.38 Ma (95% HPD, 1.82–2.93 Ma), which fits well with this timescale as well. The drastic uplift of the plateau and evolution of Asian monsoons largely re-shaped the landscape features of eastern Asia, including drainage systems [88, 89], which have been hypothesized to be important driving forces of vicariant speciation or intraspecific divergence in many fish species [3, 18, 90, 91]. Similar effects are reported for many taxa, including plants [92, 93] and other animals [12, 31, 94].

Lineages B and C, which comprised most individuals from the Yangtze River basin, separated at ~1.61 Ma (95% HPD, 1.19–1.98 Ma), which corresponds to the hypothetical formation of the current Yangtze River [95]. Historically, the Upper Yangtze River flowed southward into the Paleo-Red River [20, 96] and the middle lower Yangtze River flowed into the East China Sea [96]. Although the exact time for the connection at the Three Gorges is controversial, this appears to have occurred sometime between the Late Pliocene and Early Pleistocene, when the Yangtze River shifted its drainage network [95]. The time of splitting between the two lineages estimated in our study fits this timescale. This historical break is recorded in the level of genetic divergence between the populations of many taxa [21, 22]. Considering only Cytb was used to estimate divergence times among the lineages, our presented hypotheses should be treated cautiously.

In addition to the influence of the geologic movements, poor dispersal ability may play prominent roles in the divergence of *H. leucisculus* populations. Firstly, the lower dispersal potential inferred from Mantel analyses indicates that geographic distance acts as a barrier for gene exchange between populations and may lead to genetic divergence. Furthermore, Cytb analyses suggest that *H. leucisculus* is geographically restricted to local areas, which may be due to the poor dispersal potential. For example, approximately 78.4% of Cytb haplotypes were only detected in single locality, which mainly distrubuted in Localities 3–12 and 17–26 (Additional file 2: Table S6). The AMOVA and ɸ_ST_ calculations suggest a high level of geographic structuring. This pattern may result from the isolation of populations due to habitat restriction.

### Demographic history

Cyclical Pleistocene glaciations in Europe and North America have been demonstrated to influence the population demographic history of many extant species [23, 24]. Although it is widely acknowledged that East Asia did not undergo major glaciations during the Quaternary [26, 27], a number of studies have suggested that climatic oscillations were responsible for the genetic diversity and current distribution for some taxa [35, 97–100]. A similar scenario has been detected in some extant taxa in southern China [32–34, 100].

In our study, lineages B and C of *H. leucisculus* fit this scenario. Population demographic analyses, including a neutrality test (Fu’*s*) and EBSPs, consistently indicated that rapid population expansion has occurred in the two lineages. The absence of sublineages probably further reflects an expansion event for the two lineages. Lineage B mainly occurred in narrow regions (Localities 5–7) and exhibited low genetic diversity, which was easily influenced by fluctuant climate. In contrast, the expansion time of lineage C (~0.08 Ma) was much earlier than that of lineage B, occurring in the Late Pleistocene with the Riss-Würm interglaciation (0.073–0.125 Ma), when the climate in China was warm and wet [101, 102]. During this period, the warm climate was suitable for *H. leucisculus* survival and dispersal. Furthermore, the floods frequently occurred in summer may be a potential factor that can promote the population expansion of this species. During annual rainfall periods, the floods are common, allowing fluvial connectivity restoration and providing a contemporary condition for *H. leucisculus* population expansion. Certainly, the decreasing of competition and predation may also contribute to the population expansion of these two lineages.

However, we found no evidence of long-distance dispersal and population expansion in lineage A, which is principally distributed in southernmost China. In these temperate regions and the tropics, the relatively mild Pleistocene climate in southern China [28, 29, 103] and the varied topography provided many suitable habitats for *H. leucisculus* [23]. The suitable habitats may still have been present in these regions during the glacial periods. Without pressure from a lack of habitat, it is possible that *H. leucisculus* inhabiting these regions might have remained in stable niches and did not experience drastic demographic fluctuations. The high level of genetic diversity reflects this scenario. Similar examples were reported in other taxa in southern China [30, 31, 104].

### Implications of cryptic speciation

Many hidden species (cryptic species) with the same morphological characteristics have been recognized using molecular tools and integrative taxonomy, which enrich species diversity but challenge traditional morphological taxonomy [105]. In our study, the BPP analysis based on nuclear genes suggests that the three lineages (A, B and C) tested are valid species. The three-lineage divergence is also supported by AMOVA results (Table 2). In addition, the three mtDNA lineages could be clearly distinguished in the three nDNA gene (ENC1, RAG2 and zic1) phylogenetic trees. Consequently, the three lineages may correspond to three separate species in *H. leucisculus* under the phylogenetic species concept [106]. Thus, given that no significant morphological differentiation among these populations was revealed in a previous study [107], *H. leucisculus* may have undergone cryptic speciation. However, the formal taxonomies of the DNA-based cryptic lineages are problematic [108]. The cryptic lineages in *H. leucisculus* detected by unlinked molecular markers merely conform to the primary species hypotheses, and more morphological analysis and phylogenetic evidence are needed to clarify formal taxonomic assignments of each lineage under the criteria of secondary species hypotheses [109].

### Gene flow between lineages

IM analysis provides unambiguous evidence of gene flow from lineages B to A, from C to A, and from C to B (Fig. 5). In the three cases, the exchange is unidirectional. It is widely acknowledged that speciation with ongoing gene flow is common [110]. The detected levels of gene flow (2NM = 0.051–0.29) represent substantial gene flow but not high enough to prevent divergence; a 2NM greater than one would limit the divergence process in the absence of selection [69, 111, 112].

## Conclusions

Our study documents the phylogeographic structure and cryptic speciation of *H. leucisculus* in the drainages of southern China. Three mtDNA lineages were observed, and each lineage represents a valid species. Intense uplift of the Qinghai–Tibetan Plateau, evolution of Asian monsoons, changes in paleo-drainages, local adaptation and poor dispersal ability may have driven the divergence of the three putative species. However, gene flow occurs among the three lineages. Demographic analyses suggest that lineages B and C have experienced rapid demographic expansion, whereas lineage A remained stable long term during the cyclical Pleistocene glacial periods. Our results reveal the phylogeographical structure, cryptic diversity and evolutionary history of *H. leucisculus* populations and reinforce the notion that the number of widespread Chinese cyprinid species has been underestimated.

## Additional files

Additional file 1: Table S1. Summary of sample localities for *Hemiculter leucisculus* and outgroups. Locality numbers correspond to Fig. 1. Locality, coordinates (latitude/longitude), voucher number, and GenBank accession number for Cytb and nuclear loci are presented. Genbank numbers in bold and italic are sequences from Fan & He (2014) [1], and in bold are sequences from NCBI dataset. (DOCX 108 kb)

Additional file 2: Table S2. Information of primer pairs used in our study. **Table S3** Summary statistics for the 13 loci used in this study. bp, length of locus in bases; n, number of sequences; ps, polymorphic sites; pis, parsimony informative sites; LNR, length of the longest non-recombining regions, h, number of haplotypes. **Table S4** Nucleotide substitution models used in tree reconstruction. Pinvar, proportion of invariable sites; Gamma, gamma shape parameter. **Table S5** Nucleotide substitution models for Cytb and three nuDNA loci used in extend Bayesian skyline plots. **Table S6** Genetic diversity statistics for each population based on Cytb. n, individual numbers; Nh, haplotype numbers; ph, private haplotype within each population; Hap, Cytb haplotype; *h*, haplotype diversity; π, nucleotide diversity. **Table S7** Genetic distance based on Cytb among the three lineages estimated by K2P distance (%). **Figure S1** The entire distribution of *Hemiculter leucisculus* in global scale. The map derived from http://www.discoverlife.org. (DOCX 267 kb)
