# Supplementary material for: Phylogeographic structure, cryptic speciation and demographic history of the sharpbelly (Hemiculter leucisculus), a freshwater habitat generalist from southern China
Source: BMC Evol Biol. 2017 Sep 12;17:216. doi: 10.1186/s12862-017-1058-0 (PMC5596851; doi:10.1186/s12862-017-1058-0)
Supplement: Supplementary file 1 — Summary of sample localities for Hemiculter leucisculus and outgroups. Locality numbers correspond to Fig. 1. Locality, coordinates (latitude/longitude), voucher number, and GenBank accession number for Cytb and nuclear loci are presented. Genbank numbers in bold and italic are sequences from Fan & He (2014) [1], and in bold are sequences from NCBI dataset. (DOCX 108 kb) [file 12862_2017_1058_MOESM1_ESM.docx]

**Phylogeographic structure, cryptic speciation and demographic history of the sharpbelly (*Hemiculter leucisculus*), a freshwater habitat generalist from southern China**

Weitao Chen^1,2^, Zaixuan Zhong^1,2^, Wei Dai^1,2^, Qi Fan^1^ and Shunping He^1*^

^1^The Key Laboratory of Aquatic Biodiversity and Conservation of Chinese Academy of Sciences, Institute of Hydrobiology, Chinese Academy of Sciences, Wuhan, Hubei 430072, China

^2^University of Chinese Academy of Sciences, Beijing 100049, People’s Republic of China

**Table S1** Summary of sample localities for *Hemiculter leucisculus* and outgroups. Locality numbers correspond to Figure 1. Locality, coordinates (latitude/longitude), voucher number, and GenBank accession number for Cytb and nuclear loci are presented. Genbank numbers in bold and italic are sequences from Fan & He (2014) [1], and in bold are sequences from NCBI dataset.

| **Locality (number)** | **Lon** | **Lat** | **Drainage** | **Sample size** | **Voucher No.** |  | |  |  |  |  | **GenBank Accession Nos.** | |  |  |  |  |  |
| --- | --- | --- | --- | --- | --- | --- | --- | --- | --- | --- | --- | --- | --- | --- | --- | --- | --- | --- |
|  |  |  |  |  |  | Cytb | EGR3 | ENC1 | Glyt | myh6 | plagl2 | Ptr | RAG2 | Rhodopsin | RYR3 | SH3PX3 | sreb2 | zic1 |
|  |  |  |  |  |  |  |  |  |  |  |  |  |  |  |  |  |  |  |
| Gongguoqiao, Dali, Yunnan (1) | 99.3246 | 25.5968 | Lancangjiang R. | 4 | LA1 | KY292638 | KY292694 | KY292744 | KY292792 | KY292844 | KY292890 | KY292940 | KY292985 | KY293041 | KY293094 | KY293140 | KY293174 | KY293200 |
|  |  |  |  |  | LA2 | KY292644 |  |  |  |  |  |  |  |  |  |  |  |  |
|  |  |  |  |  | LA3 | KY292645 |  |  |  |  |  |  |  |  |  |  |  |  |
|  |  |  |  |  | LA4 | KY292646 |  |  |  |  |  |  |  |  |  |  |  |  |
| Erhai, Dali, Yunnan (2) | 100.2229 | 25.7018 | Lancangjiang R. | 9 | LA5 | KY292647 |  |  |  |  |  |  |  |  |  |  |  |  |
|  |  |  |  |  | LA6 | KY292648 |  |  |  |  |  |  |  |  |  |  |  |  |
|  |  |  |  |  | LA7 | KY292649 |  |  |  |  |  |  |  |  |  |  |  |  |
|  |  |  |  |  | LA9 | KY292650 |  |  |  |  |  |  |  |  |  |  |  |  |
|  |  |  |  |  | LA10 | KY292639 |  |  |  |  |  |  |  |  |  |  |  |  |
|  |  |  |  |  | LA16 | KY292640 |  |  |  |  |  |  |  |  |  |  |  |  |
|  |  |  |  |  | LA17 | KY292641 | KY292695 | KY292745 | KY292793 | KY292845 | KY292891 | KY292941 | KY292986 | KY293042 | KY293095 | KY293141 | KY293175 | KY293201 |
|  |  |  |  |  | LA18 | KY292642 |  |  |  |  |  |  |  |  |  |  |  |  |
|  |  |  |  |  | LA19 | KY292643 | KY292696 | KY292746 | KY292794 | KY292846 | KY292892 | KY292942 | KY292987 | KY293043 | KY293096 |  | KY293176 | KY293202 |
| Yiliang, Kunming, Yunnan (3) | 103.1574 | 24.8719 | Pearl R. | 12 | NA1 | KY292651 | KY292713 | KY292762 | KY292810 | KY292862 | KY292862 | KY292959 | KY293007 | KY293061 | KY293113 | KY293156 | KY293185 | KY293215 |
|  |  |  |  |  | NA2 | KY292657 |  |  |  |  |  |  |  |  |  |  |  |  |
|  |  |  |  |  | NA3 | KY292658 |  |  |  |  |  |  |  |  |  |  |  |  |
|  |  |  |  |  | NA4 | KY292659 |  |  |  |  |  |  |  |  |  |  |  |  |
|  |  |  |  |  | NA5 | KY292660 |  |  |  |  |  |  |  |  |  |  |  |  |
|  |  |  |  |  | NA6 | KY292661 |  |  |  |  |  |  |  |  |  |  |  |  |
|  |  |  |  |  | NA7 | KY292662 |  |  |  |  |  |  |  |  |  |  |  |  |
|  |  |  |  |  | NA11 | KY292652 |  |  |  |  |  |  |  |  |  |  |  |  |
|  |  |  |  |  | NA12 | KY292653 |  |  |  |  |  |  |  |  |  |  |  |  |
|  |  |  |  |  | NA13 | KY292654 | KY292714 | KY292763 | KY292811 | KY292863 | KY292863 | KY292960 |  | KY293062 | KY293114 | KY293157 | KY293186 | KY293215 |
|  |  |  |  |  | NA14 | KY292655 |  |  |  |  |  |  |  |  |  |  |  |  |
|  |  |  |  |  | NA16 | KY292656 |  |  |  |  |  |  |  |  |  |  |  |  |
| Wutongqiao, Leshan, Sichuan (4) | 103.8016 | 29.3856 | Yangtze R. | 19 | WTQ10 | *KF021132* |  |  |  |  |  |  |  |  |  |  |  |  |
|  |  |  |  |  | WTQ11 | *KF021133* |  |  |  |  |  |  |  |  |  |  |  |  |
|  |  |  |  |  | WTQ12 | *KF021134* |  |  |  |  |  |  |  |  |  |  |  |  |
|  |  |  |  |  | WTQ13 | *KF021135* |  |  |  |  |  |  |  |  |  |  |  |  |
|  |  |  |  |  | WTQ14 | *KF021136* | KY292715 | KY292764 | KY292812 | KY292864 | KY292864 | KY292961 |  | KY293063 | KY293115 | KY293158 |  |  |
|  |  |  |  |  | WTQ15 | *KF021137* | KY292716 | KY292765 | KY292813 | KY292865 | KY292865 | KY292962 |  | KY293064 | KY293116 | KY293159 |  |  |
|  |  |  |  |  | WTQ16 | *KF021138* |  |  |  |  |  |  |  |  |  |  |  |  |
|  |  |  |  |  | WTQ17 | *KF021139* |  |  |  |  |  |  |  |  |  |  |  |  |
|  |  |  |  |  | WTQ18 | *KF021140* |  |  |  |  |  |  |  |  |  |  |  |  |
|  |  |  |  |  | WTQ19 | *KF021141* |  |  |  |  |  |  |  |  |  |  |  |  |
|  |  |  |  |  | WTQ1 | *KF021142* |  |  |  |  |  |  |  |  |  |  |  |  |
|  |  |  |  |  | WTQ2 | *KF021143* |  |  |  |  |  |  |  |  |  |  |  |  |
|  |  |  |  |  | WTQ3 | *KF021144* |  |  |  |  |  |  |  |  |  |  |  |  |
|  |  |  |  |  | WTQ4 | *KF021145* |  |  |  |  |  |  |  |  |  |  |  |  |
|  |  |  |  |  | WTQ5 | *KF021146* |  |  |  |  |  |  |  |  |  |  |  |  |
|  |  |  |  |  | WTQ6 | *KF021147* |  |  |  |  |  |  |  |  |  |  |  |  |
|  |  |  |  |  | WTQ7 | *KF021148* |  |  |  |  |  |  |  |  |  |  |  |  |
|  |  |  |  |  | WTQ8 | *KF021149* |  |  |  |  |  |  |  |  |  |  |  |  |
| Luzhou, Sichuan (5) | 105.4069 | 28.8617 | Yangtze R. | 37 | LZ10 | *KF021040* |  |  |  |  |  |  |  |  |  |  |  |  |
|  |  |  |  |  | LZ11 | *KF021041* |  |  |  |  |  |  |  |  |  |  |  |  |
|  |  |  |  |  | LZ12 | *KF021042* |  |  |  |  |  |  |  |  |  |  |  |  |
|  |  |  |  |  | LZ13 | *KF021043* |  |  |  |  |  |  |  |  |  |  |  |  |
|  |  |  |  |  | LZ14 | *KF021044* |  |  |  |  |  |  |  |  |  |  |  |  |
|  |  |  |  |  | LZ15 | *KF021045* |  |  |  |  |  |  |  |  |  |  |  |  |
|  |  |  |  |  | LZ19 | *KF021046* |  |  |  |  |  |  |  |  |  |  |  |  |
|  |  |  |  |  | LZ1 | *KF021047* | KY292697 | KY292749 | KY292797 | KY292849 | KY292894 | KY292945 | KY292990 | KY293046 | KY293099 | KY293144 |  |  |
|  |  |  |  |  | LZ20 | *KF021048* |  |  |  |  |  |  |  |  |  |  |  |  |
|  |  |  |  |  | LZ21 | *KF021049* |  |  |  |  |  |  |  |  |  |  |  |  |
|  |  |  |  |  | LZ23 | *KF021050* |  |  |  |  |  |  |  |  |  |  |  |  |
|  |  |  |  |  | LZ25 | *KF021051* |  |  |  |  |  |  |  |  |  |  |  |  |
|  |  |  |  |  | LZ26 | *KF021052* |  |  |  |  |  |  |  |  |  |  |  |  |
|  |  |  |  |  | LZ27 | *KF021053* |  |  |  |  |  |  |  |  |  |  |  |  |
|  |  |  |  |  | LZ28 | *KF021054* |  |  |  |  |  |  |  |  |  |  |  |  |
|  |  |  |  |  | LZ29 | *KF021055* |  |  |  |  |  |  |  |  |  |  |  |  |
|  |  |  |  |  | LZ2 | *KF021056* |  |  |  |  |  |  | KY292991 |  |  |  |  |  |
|  |  |  |  |  | LZ30 | *KF021057* | KY292704 | KY292750 | KY292798 | KY292850 | KY292895 | KY292946 | KY292992 | KY293047 | KY293100 | KY293145 | KY293179 | KY293205 |
|  |  |  |  |  | LZ31 | *KF021058* | KY292705 | KY292751 | KY292799 | KY292851 | KY292896 | KY292947 |  | KY293048 | KY293101 | KY293146 | KY293180 | KY293206 |
|  |  |  |  |  | LZ32 | *KF021059* | KY292706 |  |  |  |  |  | KY292993 | KY293049 |  |  |  |  |
|  |  |  |  |  | LZ33 | *KF021060* |  |  |  |  |  |  |  |  |  | KY293147 |  |  |
|  |  |  |  |  | LZ34 | *KF021061* | KY292708 | KY292752 |  | KY292852 | KY292897 | KY292948 | KY292994 | KY293050 | KY293102 | KY293148 |  | KY293207 |
|  |  |  |  |  | LZ35 | *KF021062* |  |  |  |  |  |  |  |  |  |  |  |  |
|  |  |  |  |  | LZ36 | *KF021063* |  |  |  |  |  |  |  |  |  |  |  |  |
|  |  |  |  |  | LZ37 | *KF021064* |  |  |  |  |  |  |  |  |  |  |  |  |
|  |  |  |  |  | LZ38 | *KF021065* | KY292709 | KY292753 | KY292800 | KY292853 | KY292898 | KY292949 | KY292995 |  | KY293103 | KY293149 | KY293181 | KY293208 |
|  |  |  |  |  | LZ39 | *KF021066* |  |  |  |  |  |  |  |  |  |  |  |  |
|  |  |  |  |  | LZ3 | *KF021067* |  |  |  |  |  |  |  |  |  |  |  |  |
|  |  |  |  |  | LZ40 | *KF021068* |  |  |  |  |  |  |  |  |  |  |  |  |
|  |  |  |  |  | LZ41 | *KF021069* | KY292710 | KY292754 | KY292801 | KY292854 | KY292899 | KY292950 | KY292997 | KY293051 | KY293104 | KY293150 | KY293182 | KY293209 |
|  |  |  |  |  | LZ42 | *KF021070* |  |  |  |  |  |  |  |  |  |  |  |  |
|  |  |  |  |  | LZ4 | *KF021071* |  |  |  |  |  |  | KY292996 |  |  |  |  |  |
|  |  |  |  |  | LZ5 | *KF021072* |  |  |  |  |  |  |  |  |  |  |  |  |
|  |  |  |  |  | LZ6 | *KF021073* |  |  |  |  |  |  |  |  |  |  |  |  |
|  |  |  |  |  | LZ7 | *KF021074* | KY292711 | KY292755 | KY292802 | KY292855 | KY292900 | KY292951 | KY292998 | KY293052 | KY293105 |  |  | KY293210 |
|  |  |  |  |  | LZ8 | *KF021075* | KY292712 |  | KY292803 | KY292856 | KY292901 | KY292952 | KY292999 | KY293053 | KY293106 | KY293151 | KY293183 | KY293211 |
|  |  |  |  |  | LZ9 | *KF021076* |  |  |  |  |  |  |  |  |  |  |  |  |
| Hejiang, Luzhou, Sichuan (6) | 105.8303 | 28.8204 | Yangtze R. | 2 | HJ4 | *KF021220* | KY292698 | KY292736 | KY292785 | KY292836 |  | KY292933 | KY292980 | KY293034 | KY293086 |  |  |  |
|  |  |  |  |  | HJ5 | *KF021221* |  |  |  |  |  |  |  |  |  |  |  |  |
| Mudong, Chongqing (7) | 106.8428 | 29.5809 | Yangtze R. | 28 | MD10 | *KF021077* |  |  |  |  |  |  |  |  |  |  |  |  |
|  |  |  |  |  | MD12 | *KF021078* | KY292699 | KY292757 | KY292805 | KY292858 | KY292903 | KY292954 | KY293002 | KY293056 | KY293108 | KY293153 |  |  |
|  |  |  |  |  | MD13 | *KF021079* |  |  |  |  |  |  |  |  |  |  |  |  |
|  |  |  |  |  | MD15 | *KF021080* |  |  |  |  |  |  |  |  |  |  |  |  |
|  |  |  |  |  | MD16 | *KF021081* |  |  |  |  |  |  |  |  |  |  |  |  |
|  |  |  |  |  | MD17 | *KF021082* |  |  |  |  |  |  |  |  |  |  |  |  |
|  |  |  |  |  | MD19 | *KF021083* | KY292691 | KY292758 | KY292806 |  | KY292904 | KY292955 | KY293003 | KY293057 | KY293109 | KY293154 |  |  |
|  |  |  |  |  | MD1 | *KF021084* | KY292700 | KY292756 | KY292804 | KY292857 | KY292902 | KY292953 | KY293001 | KY293055 | KY293107 | KY293152 | KY293184 | KY293212 |
|  |  |  |  |  | MD20 | *KF021085* |  |  |  |  |  |  |  |  |  |  |  |  |
|  |  |  |  |  | MD21 | *KF021086* |  |  |  |  |  |  |  |  |  |  |  |  |
|  |  |  |  |  | MD22 | *KF021087* |  |  |  |  |  |  |  |  |  |  |  |  |
|  |  |  |  |  | MD23 | *KF021088* |  |  |  |  |  |  |  |  |  |  |  |  |
|  |  |  |  |  | MD25 | *KF021089* |  |  |  |  |  |  |  | KY293058 |  |  |  |  |
|  |  |  |  |  | MD28 | *KF021090* |  |  |  |  |  |  |  |  |  |  |  |  |
|  |  |  |  |  | MD29 | *KF021091* |  |  |  |  |  |  |  |  |  |  |  |  |
|  |  |  |  |  | MD2 | *KF021092* |  |  |  |  |  |  |  |  |  |  |  |  |
|  |  |  |  |  | MD30 | *KF021093* |  |  |  |  |  |  |  |  |  |  |  |  |
|  |  |  |  |  | MD31 | *KF021094* | KY292701 | KY292759 | KY292807 | KY292859 | KY292905 | KY292956 | KY293004 | KY293059 | KY293110 |  |  |  |
|  |  |  |  |  | MD33 | *KF021095* |  |  |  |  |  |  |  |  |  |  |  |  |
|  |  |  |  |  | MD34 | *KF021096* |  |  |  |  |  |  |  |  |  |  |  |  |
|  |  |  |  |  | MD35 | *KF021097* |  |  |  |  |  |  |  |  |  |  |  |  |
|  |  |  |  |  | MD36 | *KF021098* |  |  |  |  |  |  |  |  |  |  |  |  |
|  |  |  |  |  | MD3 | *KF021099* |  |  |  |  |  |  |  |  |  |  |  |  |
|  |  |  |  |  | MD4 | *KF021100* |  |  |  |  |  |  |  |  |  |  |  |  |
|  |  |  |  |  | MD5 | *KF021101* | KY292702 | KY292760 | KY292808 | KY292860 | KY292906 | KY292957 | KY293005 |  | KY293111 | KY293155 |  | KY293213 |
|  |  |  |  |  | MD6 | *KF021102* |  |  |  |  |  |  |  |  |  |  |  |  |
|  |  |  |  |  | MD8 | *KF021103* |  |  |  |  |  |  |  |  |  |  |  |  |
|  |  |  |  |  | MD9 | *KF021104* | KY292703 | KY292761 | KY292809 | KY292861 | KY292907 | KY292958 | KY293006 | KY293060 | KY293112 |  |  | KY293214 |
| Tian’e, Hechi, Guangxi (8) | 107.1792 | 24.9815 | Pearl R. | 13 | TE10 | KY292625 |  |  |  |  |  |  |  |  |  |  |  |  |
|  |  |  |  |  | TE11 | KY292626 |  |  |  |  |  |  |  |  |  |  |  |  |
|  |  |  |  |  | TE12 | KY292627 |  |  |  |  |  |  |  |  |  |  |  |  |
|  |  |  |  |  | TE13 | KY292628 |  |  |  |  |  |  |  |  |  |  |  |  |
|  |  |  |  |  | TE14 | KY292629 |  |  |  |  |  |  |  |  |  |  |  |  |
|  |  |  |  |  | TE18 | KY292630 |  |  |  |  |  |  |  |  |  |  |  |  |
|  |  |  |  |  | TE2 | KY292631 |  |  |  |  |  |  |  |  |  |  |  |  |
|  |  |  |  |  | TE4 | KY292632 |  |  |  |  |  |  |  |  |  |  |  |  |
|  |  |  |  |  | TE5 | KY292633 |  |  |  |  |  |  |  |  |  |  |  |  |
|  |  |  |  |  | TE6 | KY292634 |  |  |  |  |  |  | KY293009 |  |  |  |  |  |
|  |  |  |  |  | TE7 | KY292635 |  |  |  |  |  |  |  |  |  |  |  |  |
|  |  |  |  |  | TE8 | KY292636 |  |  |  |  |  |  |  |  |  |  |  |  |
|  |  |  |  |  | TE9 | KY292637 |  |  |  |  |  |  |  |  |  |  |  |  |
| Qinjiang, Guangxi (9) |  |  | Pearl R. | 1 |  | **AY089711^2^** |  |  |  |  |  |  |  |  |  |  |  |  |
| Hengxian, Nanning, Guangxi (10) | 109.2568 | 22.6811 | Pearl R. | 36 | HX10 | KY292574 |  |  |  |  |  |  |  |  |  |  |  |  |
|  |  |  |  |  | HX11 | KY292575 |  |  |  |  |  |  |  |  |  |  |  |  |
|  |  |  |  |  | HX12 | KY292576 | KY292669 | KY292738 | KY292787 | KY292838 | KY292884 | KY292935 | KY292982 | KY293036 | KY293088 | KY293135 | KY293170 | KY293196 |
|  |  |  |  |  | HX13 | KY292577 |  |  |  |  |  |  |  |  |  |  |  |  |
|  |  |  |  |  | HX14 | KY292578 |  |  |  |  |  |  |  |  |  |  |  |  |
|  |  |  |  |  | HX15 | KY292579 |  |  |  |  |  |  |  |  |  |  |  |  |
|  |  |  |  |  | HX16 | KY292580 |  |  |  |  |  |  |  |  |  |  |  |  |
|  |  |  |  |  | HX17 | KY292581 |  |  |  |  |  |  |  |  |  |  |  |  |
|  |  |  |  |  | HX18 | KY292582 |  |  |  |  |  |  |  |  |  |  |  |  |
|  |  |  |  |  | HX19 | KY292583 |  |  |  |  |  |  |  |  |  |  |  |  |
|  |  |  |  |  | HX1 | KY292584 |  |  |  |  |  |  |  |  |  |  |  |  |
|  |  |  |  |  | HX20 | KY292585 |  |  |  |  |  |  |  |  |  |  |  |  |
|  |  |  |  |  | HX21 | KY292586 |  |  |  |  |  |  |  |  |  |  |  |  |
|  |  |  |  |  | HX22 | KY292587 |  |  |  |  |  |  |  |  |  |  |  |  |
|  |  |  |  |  | HX24 | KY292588 |  |  |  |  |  |  |  |  |  |  |  |  |
|  |  |  |  |  | HX25 | KY292589 |  |  |  |  |  |  |  |  |  |  |  |  |
|  |  |  |  |  | HX26 | KY292590 |  |  |  |  |  |  |  |  |  |  |  |  |
|  |  |  |  |  | HX27 | KY292591 |  |  |  |  |  |  |  |  |  |  |  |  |
|  |  |  |  |  | HX28 | KY292592 | KY292666 | KY292739 | KY292788 | KY292839 | KY292885 | KY292936 | KY292983 | KY293037 | KY293089 | KY293136 |  | KY293197 |
|  |  |  |  |  | HX29 | KY292593 |  |  |  |  |  |  |  |  |  |  |  |  |
|  |  |  |  |  | HX2 | KY292594 |  |  |  |  |  |  |  |  |  |  |  |  |
|  |  |  |  |  | HX30 | KY292595 |  |  |  |  |  |  |  |  |  |  |  |  |
|  |  |  |  |  | HX31 | KY292596 |  |  |  |  |  |  |  |  |  |  |  |  |
|  |  |  |  |  | HX32 | KY292597 | KY292668 | KY292740 | KY292789 | KY292840 | KY292886 | KY292937 | KY292984 | KY293038 | KY293090 | KY293137 | KY293171 |  |
|  |  |  |  |  | HX33 | KY292598 |  |  |  |  |  |  |  |  |  |  |  |  |
|  |  |  |  |  | HX34 | KY292599 |  |  |  |  |  |  |  |  |  |  |  |  |
|  |  |  |  |  | HX35 | KY292600 |  |  |  |  |  |  |  |  |  |  |  |  |
|  |  |  |  |  | HX36 | KY292601 |  |  |  |  |  |  |  |  |  |  |  |  |
|  |  |  |  |  | HX37 | KY292602 |  |  |  |  |  |  |  |  |  |  |  |  |
|  |  |  |  |  | HX3 | KY292603 |  |  |  |  |  |  |  |  |  |  |  |  |
|  |  |  |  |  | HX4 | KY292604 |  |  |  |  |  |  |  |  |  |  |  |  |
|  |  |  |  |  | HX5 | KY292605 |  |  |  |  |  |  |  |  |  |  |  |  |
|  |  |  |  |  | HX6 | KY292606 |  |  |  |  |  |  |  |  |  |  |  |  |
|  |  |  |  |  | HX7 | KY292607 |  |  |  |  |  |  |  |  |  |  |  |  |
|  |  |  |  |  | HX8 | KY292608 |  |  |  |  |  |  |  |  |  |  |  |  |
|  |  |  |  |  | HX9 | KY292609 |  |  |  |  |  |  |  |  |  |  |  |  |
| Lingshan, Qinzhou, Guangxi (11) | 109.2787 | 22.4163 | Pearl R. | 15 | LS10 | KY292610 |  |  |  |  |  |  |  |  |  |  |  |  |
|  |  |  |  |  | LS11 | KY292611 |  |  |  |  |  |  |  |  |  |  |  |  |
|  |  |  |  |  | LS12 | KY292612 |  |  |  |  |  |  |  |  |  |  |  |  |
|  |  |  |  |  | LS14 | KY292613 | KY292665 | KY292747 | KY292795 | KY292847 |  | KY292943 | KY292988 | KY293044 | KY293097 | KY293142 | KY293177 | KY293203 |
|  |  |  |  |  | LS15 | KY292614 |  |  |  |  |  |  |  |  |  |  |  |  |
|  |  |  |  |  | LS16 | KY292615 | KY292671 | KY292748 | KY292796 | KY292848 | KY292893 | KY292944 | KY292989 | KY293045 | KY293098 | KY293143 | KY293178 | KY293204 |
|  |  |  |  |  | LS17 | KY292616 |  |  |  |  |  |  |  |  |  |  |  |  |
|  |  |  |  |  | LS1 | KY292617 |  |  |  |  |  |  |  |  |  |  |  |  |
|  |  |  |  |  | LS2 | KY292618 |  |  |  |  |  |  |  |  |  |  |  |  |
|  |  |  |  |  | LS3 | KY292619 |  |  |  |  |  |  |  |  |  |  |  |  |
|  |  |  |  |  | LS4 | KY292620 |  |  |  |  |  |  |  |  |  |  |  |  |
|  |  |  |  |  | LS5 | KY292621 |  |  |  |  |  |  |  |  |  |  |  |  |
|  |  |  |  |  | LS6 | KY292622 |  |  |  |  |  |  |  |  |  |  |  |  |
|  |  |  |  |  | LS7 | KY292623 |  |  |  |  |  |  |  |  |  |  |  |  |
|  |  |  |  |  | LS8 | KY292624 |  |  |  |  |  |  |  |  |  |  |  |  |
| Bobai, Yulin, Guangxi (12) | 109.9144 | 22.2344 | Pearl R. | 9 | BB10 | KY292565 |  |  |  |  |  |  |  |  |  |  |  |  |
|  |  |  |  |  | BB11 | KY292566 |  |  |  |  |  |  |  |  |  |  |  |  |
|  |  |  |  |  | BB1 | KY292567 | KY292664 | KY292720 | KY292772 | KY292820 | KY292872 | KY292916 | KY292968 | KY293015 | KY293070 |  | KY293164 |  |
|  |  |  |  |  | BB2 | KY292573 | KY292667 | KY292721 |  | KY292821 | KY292873 | KY292917 |  | KY293016 | KY293071 |  |  | KY293191 |
|  |  |  |  |  | BB3 | KY292568 |  |  |  |  |  |  |  |  |  |  |  |  |
|  |  |  |  |  | BB4 | KY292569 |  |  |  |  |  |  |  |  |  |  |  |  |
|  |  |  |  |  | BB5 | KY292570 |  |  |  |  |  |  |  |  |  |  |  |  |
|  |  |  |  |  | BB6 | KY292571 | KY292670 | KY292722 | KY292773 | KY292822 | KY292874 | KY292918 | KY292969 | KY293017 | KY293072 | KY293123 |  | KY293192 |
|  |  |  |  |  | BB7 | KY292572 |  |  |  |  |  |  |  |  |  |  |  |  |
| Nandujiang, Hainan (13) |  |  | Nanhai R. | 1 |  | **AY089709^2^** |  |  |  |  |  |  |  |  |  |  |  |  |
| Wanquanhe, Hainan (14) |  |  | Nanhai R. | 1 |  | **AY089710^2^** |  |  |  |  |  |  |  |  |  |  |  |  |
| Zhujiang, unknown (15) |  |  | Pearl R. | 1 |  | **AY089713^2^** |  |  |  |  |  |  |  |  |  |  |  |  |
| Zigui, Yichang, Hubei (16) | 110.9863 | 30.8439 | Yangtze R. | 1 | ZG36 | ***KF021210*** | KY292684 | KY292770 | KY292818 | KY292870 | KY292870 | KY292966 | KY293013 | KY293068 | KY293121 |  |  |  |
| Dongting Lake, Yueyang, Hunan (17) | 112.8928 | 29.2669 | Yangtze R. | 30 | DT10 | ***KF020928*** |  |  |  |  |  |  |  |  |  |  |  |  |
|  |  |  |  |  | DT11 | ***KF020929*** |  |  |  |  |  |  |  |  |  |  |  |  |
|  |  |  |  |  | DT12 | ***KF020930*** |  |  |  |  |  |  |  |  |  |  |  |  |
|  |  |  |  |  | DT13 | ***KF020931*** |  |  |  |  |  |  |  |  |  |  |  |  |
|  |  |  |  |  | DT15 | ***KF020932*** |  |  |  |  |  |  |  |  |  |  |  |  |
|  |  |  |  |  | DT16 | ***KF020933*** |  |  |  |  |  |  |  |  |  |  |  |  |
|  |  |  |  |  | DT17 | ***KF020934*** |  |  |  |  |  |  |  |  |  |  |  |  |
|  |  |  |  |  | DT18 | ***KF020935*** |  |  |  |  |  |  |  |  |  |  |  |  |
|  |  |  |  |  | DT1 | ***KF020936*** |  |  |  |  |  |  |  |  |  |  |  |  |
|  |  |  |  |  | DT20 | ***KF020937*** |  |  |  |  |  |  |  |  |  |  |  |  |
|  |  |  |  |  | DT21 | ***KF020938*** |  |  |  |  |  |  |  |  |  |  |  |  |
|  |  |  |  |  | DT22 | ***KF020939*** |  |  |  |  |  |  |  |  |  |  |  |  |
|  |  |  |  |  | DT23 | ***KF020940*** |  |  |  |  |  |  |  |  |  |  |  |  |
|  |  |  |  |  | DT24 | ***KF020941*** |  |  |  |  |  |  |  |  |  |  |  |  |
|  |  |  |  |  | DT25 | ***KF020942*** |  |  |  |  |  |  |  |  |  |  |  |  |
|  |  |  |  |  | DT26 | ***KF020943*** |  |  |  |  |  |  |  |  |  |  |  |  |
|  |  |  |  |  | DT27 | ***KF020944*** |  |  |  |  |  |  |  |  |  |  |  |  |
|  |  |  |  |  | DT28 | ***KF020945*** |  |  |  |  |  |  |  |  |  |  |  |  |
|  |  |  |  |  | DT29 | ***KF020946*** | KY292676 | KY292735 |  | KY292835 | KY292882 | KY292932 | KY292978 | KY293033 | KY293085 | KY293133 |  |  |
|  |  |  |  |  | DT2 | ***KF020947*** |  |  |  |  |  |  |  |  |  |  |  |  |
|  |  |  |  |  | DT30 | ***KF020948*** |  |  |  |  |  |  |  |  |  |  |  |  |
|  |  |  |  |  | DT31 | ***KF020949*** |  |  |  |  |  |  |  |  |  |  |  |  |
|  |  |  |  |  | DT32 | ***KF020950*** |  |  |  |  |  |  |  |  |  |  |  |  |
|  |  |  |  |  | DT33 | ***KF020951*** |  |  |  |  |  |  |  |  |  |  |  |  |
|  |  |  |  |  | DT34 | ***KF020952*** |  |  |  |  |  |  |  |  |  |  |  |  |
|  |  |  |  |  | DT3 | ***KF020953*** |  |  |  |  |  |  |  |  |  |  |  |  |
|  |  |  |  |  | DT5 | ***KF020954*** |  |  |  |  |  |  |  |  |  |  |  |  |
|  |  |  |  |  | DT7 | ***KF020955*** |  |  |  |  |  |  |  |  |  |  |  |  |
|  |  |  |  |  | DT8 | ***KF020956*** |  |  |  |  |  |  |  |  |  |  |  |  |
|  |  |  |  |  | DT9 | ***KF020957*** |  |  |  |  |  |  |  |  |  |  |  |  |
| Honghu Lake, Jingzhou, Hubei (18) | 113.3184 | 29.8130 | Yangtze R. | 26 | HH11 | ***KF020958*** |  |  |  |  |  |  |  |  |  |  |  |  |
|  |  |  |  |  | HH12 | ***KF020959*** |  |  |  |  |  |  |  |  |  |  |  |  |
|  |  |  |  |  | HH13 | ***KF020960*** |  |  |  |  |  |  |  |  |  |  |  |  |
|  |  |  |  |  | HH15 | ***KF020961*** |  |  |  |  |  |  |  |  |  |  |  |  |
|  |  |  |  |  | HH16 | ***KF020962*** |  |  |  |  |  |  |  |  |  |  |  |  |
|  |  |  |  |  | HH17 | ***KF020963*** |  |  |  |  |  |  |  |  |  |  |  |  |
|  |  |  |  |  | HH18 | ***KF020964*** |  |  |  |  |  |  |  |  |  |  |  |  |
|  |  |  |  |  | HH1 | ***KF020965*** |  |  |  |  |  |  |  |  |  |  |  |  |
|  |  |  |  |  | HH21 | ***KF020966*** |  |  |  |  |  |  |  |  |  |  |  |  |
|  |  |  |  |  | HH22 | ***KF020967*** |  |  |  |  |  |  |  |  |  |  |  |  |
|  |  |  |  |  | HH23 | ***KF020968*** |  |  |  |  |  |  |  |  |  |  |  |  |
|  |  |  |  |  | HH24 | ***KF020969*** |  |  |  |  |  |  |  |  |  |  |  |  |
|  |  |  |  |  | HH25 | ***KF020970*** |  |  |  |  |  |  |  |  |  |  |  |  |
|  |  |  |  |  | HH26 | ***KF020971*** |  |  |  |  |  |  |  |  |  |  |  |  |
|  |  |  |  |  | HH27 | ***KF020972*** |  |  |  |  |  |  |  |  |  |  |  |  |
|  |  |  |  |  | HH28 | ***KF020973*** |  |  |  |  |  |  |  |  |  |  |  |  |
|  |  |  |  |  | HH29 | ***KF020974*** |  |  |  |  |  |  |  |  |  |  |  |  |
|  |  |  |  |  | HH2 | ***KF020975*** |  |  |  |  |  |  |  |  |  |  |  |  |
|  |  |  |  |  | HH30 | ***KF020976*** |  |  |  |  |  |  |  |  |  |  |  |  |
|  |  |  |  |  | HH31 | ***KF020977*** |  |  |  |  |  |  |  |  |  |  |  |  |
|  |  |  |  |  | HH3 | ***KF020978*** |  |  |  |  |  |  |  |  |  |  |  |  |
|  |  |  |  |  | HH4 | ***KF020979*** |  |  |  |  |  |  |  |  |  |  |  |  |
|  |  |  |  |  | HH5 | ***KF020980*** |  |  |  |  |  |  |  |  |  |  |  |  |
|  |  |  |  |  | HH6 | ***KF020981*** |  |  |  |  |  |  | KY292979 |  |  |  |  |  |
|  |  |  |  |  | HH8 | ***KF020982*** |  |  |  |  |  |  |  |  |  |  |  |  |
|  |  |  |  |  | HH9 | ***KF020983*** |  |  |  |  |  |  |  |  |  |  |  |  |
| Jiayu, Xianning, Hubei (19) | 113.8825 | 30.1869 | Yangtze R. | 2 | JY1 | ***KF020984*** |  |  |  |  |  |  |  |  |  |  |  |  |
|  |  |  |  |  | JY2 | ***KF020985*** |  |  |  |  |  |  |  |  |  |  |  |  |
| Jingkou, Wuhan, Hebei (20) | 114.1151 | 30.3302 | Yangtze R. | 10 | JK11 | ***KF020989*** | KY292686 | KY292741 | KY292790 | KY292841 | KY292887 | KY292938 |  |  | KY293091 |  |  |  |
|  |  |  |  |  | JK18 | ***KF020990*** |  |  |  |  |  |  |  |  |  |  |  |  |
|  |  |  |  |  | JK1 | ***KF020991*** |  |  |  |  |  |  |  |  |  |  |  |  |
|  |  |  |  |  | JK2 | ***KF020992*** |  |  |  |  |  |  |  |  |  |  |  |  |
|  |  |  |  |  | JK3 | ***KF020993*** |  |  |  |  |  |  |  |  |  |  |  |  |
|  |  |  |  |  | JK46 | ***KF020994*** | KY292689 | KY292742 | KY292791 | KY292842 | KY292888 |  |  | KY293039 | KY293092 | KY293138 | KY293172 | KY293198 |
|  |  |  |  |  | JK4 | ***KF020995*** |  |  |  |  |  |  |  |  |  |  |  |  |
|  |  |  |  |  | JK5 | ***KF020996*** |  |  |  |  |  |  |  |  |  |  |  |  |
|  |  |  |  |  | JK6 | ***KF020997*** |  |  |  |  |  |  |  |  |  |  |  |  |
|  |  |  |  |  | JK9 | ***KF020998*** | KY292679 | KY292743 |  | KY292843 | KY292889 | KY292939 |  | KY293040 | KY293093 | KY293139 | KY293173 | KY293199 |
| Donghu, Wuhan, Hubei (21) | 114.3803 | 30.5537 | Yangtze R. | 29 | DH10 | ***KF020899*** | KY292675 | KY292728 | KY292778 | KY292827 | KY292878 | KY292924 | KY292972 | KY293023 | KY293078 | KY293128 |  |  |
|  |  |  |  |  | DH11 | ***KF020900*** |  |  |  |  |  |  |  |  |  |  |  |  |
|  |  |  |  |  | DH12 | ***KF020901*** | KY292677 |  |  |  |  |  |  | KY293024 |  |  |  |  |
|  |  |  |  |  | DH13 | ***KF020902*** |  |  |  |  |  |  |  |  |  |  |  |  |
|  |  |  |  |  | DH14 | ***KF020903*** | KY292678 | KY292729 | KY292779 | KY292828 |  | KY292925 | KY292973 | KY293025 | KY293079 |  |  |  |
|  |  |  |  |  | DH15 | ***KF020904*** |  |  |  |  |  |  |  |  |  |  |  |  |
|  |  |  |  |  | DH16 | ***KF020905*** |  |  |  |  |  |  |  |  |  |  |  |  |
|  |  |  |  |  | DH17 | ***KF020906*** |  |  |  |  |  |  |  |  |  |  |  |  |
|  |  |  |  |  | DH18 | ***KF020907*** |  |  |  |  |  |  |  |  |  |  |  |  |
|  |  |  |  |  | DH19 | ***KF020908*** | KY292682 |  |  |  |  |  | KY292974 | KY293026 |  |  |  |  |
|  |  |  |  |  | DH1 | ***KF020909*** |  |  |  |  |  |  |  |  |  |  |  |  |
|  |  |  |  |  | DH20 | ***KF020910*** | KY292683 | KY292730 | KY292780 | KY292829 | KY292879 | KY292926 | KY292975 | KY293027 | KY293080 | KY293129 | KY293167 | KY293194 |
|  |  |  |  |  | DH21 | ***KF020911*** |  |  |  |  |  |  |  |  |  |  |  |  |
|  |  |  |  |  | DH22 | ***KF020912*** |  |  |  |  |  |  |  |  |  |  |  |  |
|  |  |  |  |  | DH23 | ***KF020913*** |  |  |  |  |  |  |  |  |  |  |  |  |
|  |  |  |  |  | DH24 | ***KF020914*** |  |  |  |  |  |  |  | KY293028 |  |  |  |  |
|  |  |  |  |  | DH25 | ***KF020915*** |  |  |  |  |  |  |  |  |  |  |  |  |
|  |  |  |  |  | DH26 | ***KF020916*** | KY292687 | KY292731 | KY292781 | KY292830 | KY292880 | KY292927 |  | KY293029 | KY293081 | KY293130 | KY293168 | KY293195 |
|  |  |  |  |  | DH28 | ***KF020917*** |  |  |  |  |  |  |  |  |  |  |  |  |
|  |  |  |  |  | DH29 | ***KF020918*** | KY292688 | KY292732 | KY292782 | KY292831 |  | KY292928 |  | KY293030 | KY293082 | KY293131 |  |  |
|  |  |  |  |  | DH2 | ***KF020919*** |  |  |  |  |  |  |  |  |  |  |  |  |
|  |  |  |  |  | DH30 | ***KF020920*** |  |  |  |  |  | KY292929 |  |  |  |  |  |  |
|  |  |  |  |  | DH3 | ***KF020921*** |  |  |  |  |  |  |  |  |  |  |  |  |
|  |  |  |  |  | DH4 | ***KF020922*** |  |  |  |  |  |  |  |  |  |  |  |  |
|  |  |  |  |  | DH5 | ***KF020923*** |  |  |  |  |  |  |  |  |  |  |  |  |
|  |  |  |  |  | DH6 | ***KF020924*** | KY292690 | KY292733 | KY292783 | KY292832 | KY292881 | KY292930 | KY292976 | KY293031 | KY293083 | KY293132 | KY293169 |  |
|  |  |  |  |  | DH7 | ***KF020925*** |  |  |  | KY292833 |  |  |  |  |  |  |  |  |
|  |  |  |  |  | DH8 | ***KF020926*** |  |  |  |  |  |  |  |  |  |  |  |  |
|  |  |  |  |  | DH9 | ***KF020927*** | KY292692 | KY292734 | KY292784 | KY292834 |  | KY292931 | KY292977 | KY293032 | KY293084 |  |  |  |
| Liangzhihu Lake, Ezhou, Hubei (22) | 114.5099 | 30.2307 | Yangtze R. | 41 | LZH11 | ***KF020999*** |  |  |  |  |  |  |  |  |  |  |  |  |
|  |  |  |  |  | LZH12 | ***KF021000*** |  |  |  |  |  |  |  |  |  |  |  |  |
|  |  |  |  |  | LZH13 | ***KF021001*** |  |  |  |  |  |  |  |  |  |  |  |  |
|  |  |  |  |  | LZH14 | ***KF021002*** |  |  |  |  |  |  |  |  |  |  |  |  |
|  |  |  |  |  | LZH15 | ***KF021003*** |  |  |  |  |  |  |  |  |  |  |  |  |
|  |  |  |  |  | LZH16 | ***KF021004*** |  |  |  |  |  |  |  |  |  |  |  |  |
|  |  |  |  |  | LZH18 | ***KF021005*** |  |  |  |  |  |  |  |  |  |  |  |  |
|  |  |  |  |  | LZH19 | ***KF021006*** |  |  |  |  |  |  |  |  |  |  |  |  |
|  |  |  |  |  | LZH1 | ***KF021007*** |  |  |  |  |  |  |  |  |  |  |  |  |
|  |  |  |  |  | LZH20 | ***KF021008*** |  |  |  |  |  |  |  |  |  |  |  |  |
|  |  |  |  |  | LZH22 | ***KF021009*** |  |  |  |  |  |  |  |  |  |  |  |  |
|  |  |  |  |  | LZH24 | ***KF021010*** |  |  |  |  |  |  |  |  |  |  |  |  |
|  |  |  |  |  | LZH25 | ***KF021011*** |  |  |  |  |  |  |  |  |  |  |  |  |
|  |  |  |  |  | LZH26 | ***KF021012*** |  |  |  |  |  |  |  |  |  |  |  |  |
|  |  |  |  |  | LZH27 | ***KF021013*** |  |  |  |  |  |  |  |  |  |  |  |  |
|  |  |  |  |  | LZH2 | ***KF021014*** |  |  |  |  |  |  |  |  |  |  |  |  |
|  |  |  |  |  | LZH31 | ***KF021015*** |  |  |  |  |  |  |  |  |  |  |  |  |
|  |  |  |  |  | LZH33 | ***KF021016*** |  |  |  |  |  |  |  |  |  |  |  |  |
|  |  |  |  |  | LZH34 | ***KF021017*** |  |  |  |  |  |  |  |  |  |  |  |  |
|  |  |  |  |  | LZH37 | ***KF021018*** |  |  |  |  |  |  |  |  |  |  |  |  |
|  |  |  |  |  | LZH38 | ***KF021019*** |  |  |  |  |  |  |  |  |  |  |  |  |
|  |  |  |  |  | LZH39 | ***KF021020*** |  |  |  |  |  |  |  |  |  |  |  |  |
|  |  |  |  |  | LZH3 | ***KF021021*** |  |  |  |  |  |  |  |  |  |  |  |  |
|  |  |  |  |  | LZH40 | ***KF021022*** |  |  |  |  |  |  |  |  |  |  |  |  |
|  |  |  |  |  | LZH41 | ***KF021023*** |  |  |  |  |  |  |  |  |  |  |  |  |
|  |  |  |  |  | LZH42 | ***KF021024*** |  |  |  |  |  |  |  |  |  |  |  |  |
|  |  |  |  |  | LZH43 | ***KF021025*** |  |  |  |  |  |  |  |  |  |  |  |  |
|  |  |  |  |  | LZH44 | ***KF021026*** |  |  |  |  |  |  |  |  |  |  |  |  |
|  |  |  |  |  | LZH45 | ***KF021027*** |  |  |  |  |  |  |  |  |  |  |  |  |
|  |  |  |  |  | LZH46 | ***KF021028*** |  |  |  |  |  |  |  |  |  |  |  |  |
|  |  |  |  |  | LZH47 | ***KF021029*** |  |  |  |  |  |  |  |  |  |  |  |  |
|  |  |  |  |  | LZH48 | ***KF021030*** |  |  |  |  |  |  |  |  |  |  |  |  |
|  |  |  |  |  | LZH49 | ***KF021031*** |  |  |  |  |  |  |  |  |  |  |  |  |
|  |  |  |  |  | LZH4 | ***KF021032*** |  |  |  |  |  |  |  |  |  |  |  |  |
|  |  |  |  |  | LZH50 | ***KF021033*** |  |  |  |  |  |  |  |  |  |  |  |  |
|  |  |  |  |  | LZH51 | ***KF021034*** |  |  |  |  |  |  |  |  |  |  |  |  |
|  |  |  |  |  | LZH53 | ***KF021035*** |  |  |  |  |  |  |  |  |  |  |  |  |
|  |  |  |  |  | LZH54 | ***KF021036*** | KY292680 |  |  |  |  |  | KY293000 | KY293054 |  |  |  |  |
|  |  |  |  |  | LZH6 | ***KF021037*** |  |  |  |  |  |  |  |  |  |  |  |  |
|  |  |  |  |  | LZH7 | ***KF021038*** |  |  |  |  |  |  |  |  |  |  |  |  |
|  |  |  |  |  | LZH9 | ***KF021039*** |  |  |  |  |  |  |  |  |  |  |  |  |
| Wuxue, Huanggang, Hubei (23) | 115.5730 | 29.8378 | Yangtze R. | 31 | WX10 | ***KF021151*** |  |  |  |  |  |  |  |  |  |  |  |  |
|  |  |  |  |  | WX11 | ***KF021152*** |  |  |  |  |  |  |  |  |  |  |  |  |
|  |  |  |  |  | WX12 | ***KF021153*** |  |  |  |  |  |  |  |  |  |  |  |  |
|  |  |  |  |  | WX13 | ***KF021154*** |  |  |  |  |  |  |  |  |  |  |  |  |
|  |  |  |  |  | WX14 | ***KF021155*** |  |  |  |  |  |  |  |  |  |  |  |  |
|  |  |  |  |  | WX15 | ***KF021156*** |  |  |  |  |  |  |  |  |  |  |  |  |
|  |  |  |  |  | WX16 | ***KF021157*** |  | KY292767 | KY292815 | KY292867 | KY292867 |  | KY293011 |  | KY293118 |  |  |  |
|  |  |  |  |  | WX17 | ***KF021158*** |  |  |  |  |  |  |  |  |  |  |  |  |
|  |  |  |  |  | WX18 | ***KF021159*** |  |  |  |  |  |  |  |  |  |  |  |  |
|  |  |  |  |  | WX19 | ***KF021160*** |  | KY292768 |  |  |  |  |  |  |  |  |  |  |
|  |  |  |  |  | WX1 | ***KF021161*** |  |  |  |  |  |  |  |  |  |  |  |  |
|  |  |  |  |  | WX20 | ***KF021162*** |  |  |  |  |  |  |  |  |  |  |  |  |
|  |  |  |  |  | WX21 | ***KF021163*** |  |  |  |  |  |  |  |  |  |  |  |  |
|  |  |  |  |  | WX22 | ***KF021164*** |  |  |  |  |  |  |  |  |  |  |  |  |
|  |  |  |  |  | WX23 | ***KF021165*** |  |  |  |  |  |  |  |  |  |  |  |  |
|  |  |  |  |  | WX24 | ***KF021166*** |  |  |  |  |  |  |  |  |  |  |  |  |
|  |  |  |  |  | WX26 | ***KF021167*** |  |  |  |  |  |  |  |  |  |  |  |  |
|  |  |  |  |  | WX27 | ***KF021168*** |  |  |  |  |  |  |  |  |  |  |  |  |
|  |  |  |  |  | WX2 | ***KF021169*** |  |  |  |  |  |  |  |  |  |  |  |  |
|  |  |  |  |  | WX30 | ***KF021170*** |  |  |  |  |  |  |  |  |  |  |  |  |
|  |  |  |  |  | WX31 | ***KF021171*** |  |  |  |  |  |  |  |  |  |  |  |  |
|  |  |  |  |  | WX32 | ***KF021172*** |  |  |  |  |  |  |  |  |  |  |  |  |
|  |  |  |  |  | WX33 | ***KF021173*** |  |  |  |  |  |  |  |  |  |  |  |  |
|  |  |  |  |  | WX35 | ***KF021174*** |  |  |  |  |  |  |  |  |  |  |  |  |
|  |  |  |  |  | WX3 | ***KF021175*** |  |  |  |  |  |  |  |  |  |  |  |  |
|  |  |  |  |  | WX4 | ***KF021176*** |  |  |  |  |  |  |  |  |  |  |  |  |
|  |  |  |  |  | WX5 | ***KF021177*** |  |  |  |  |  |  |  |  |  |  |  |  |
|  |  |  |  |  | WX6 | ***KF021178*** |  |  |  |  |  |  |  |  |  |  |  |  |
|  |  |  |  |  | WX7 | ***KF021179*** |  |  |  |  |  |  |  |  |  |  |  |  |
|  |  |  |  |  | WX8 | ***KF021180*** |  |  |  |  |  |  |  |  |  |  |  |  |
|  |  |  |  |  | WX9 | ***KF021181*** | KY292681 |  | KY292816 | KY292868 | KY292868 | KY292964 |  | KY293066 | KY293119 | KY293161 | KY293188 |  |
| Xingzi, Jiujiang, Jiangxi (24) | 116.0415 | 29.4430 | Yangtze R. | 1 | XZ1 | ***KF021131*** | KY292718 | KY292769 | KY292817 | KY292869 | KY292869 | KY292965 | KY293012 | KY293067 | KY293120 | KY293162 | KY293189 |  |
| Duchang, Jiujiang, Jiangxi (25) | 116.2002 | 29.2389 | Yangtze R. | 20 | DC10 | ***KF021105*** |  |  |  |  |  |  |  |  |  |  |  |  |
|  |  |  |  |  | DC11 | ***KF021106*** |  |  |  |  |  |  |  |  |  |  |  |  |
|  |  |  |  |  | DC12 | ***KF021107*** | KY292672 | KY292724 |  |  |  | KY292920 |  | KY293019 | KY293074 | KY293125 |  |  |
|  |  |  |  |  | DC13 | ***KF021108*** |  |  |  |  |  |  |  |  |  |  |  |  |
|  |  |  |  |  | DC14 | ***KF021109*** |  |  |  |  |  |  |  |  |  |  |  |  |
|  |  |  |  |  | DC15 | ***KF021110*** |  |  |  |  |  |  |  |  |  |  |  |  |
|  |  |  |  |  | DC16 | ***KF021111*** |  |  |  |  |  |  |  |  |  |  |  |  |
|  |  |  |  |  | DC17 | ***KF021112*** |  |  |  |  |  |  |  |  |  |  |  |  |
|  |  |  |  |  | DC18 | ***KF021113*** |  |  |  |  |  |  |  |  |  |  |  |  |
|  |  |  |  |  | DC19 | ***KF021114*** | KY292707 | KY292725 | KY292775 | KY292824 | KY292876 | KY292921 | KY292971 | KY293020 | KY293075 |  |  | KY293193 |
|  |  |  |  |  | DC1 | ***KF021115*** | KY292685 | KY292723 | KY292774 | KY292823 | KY292875 | KY292919 | KY292970 | KY293018 | KY293073 | KY293124 | KY293165 |  |
|  |  |  |  |  | DC20 | ***KF021116*** |  |  |  |  |  |  |  |  |  |  |  |  |
|  |  |  |  |  | DC2 | ***KF021117*** |  |  |  |  |  |  |  |  |  |  |  |  |
|  |  |  |  |  | DC3 | ***KF021118*** |  |  |  |  |  |  |  |  |  |  |  |  |
|  |  |  |  |  | DC4 | ***KF021119*** |  |  |  |  |  |  |  |  |  |  |  |  |
|  |  |  |  |  | DC5 | ***KF021120*** | KY292673 | KY292726 | KY292776 | KY292825 |  | KY292922 |  | KY293021 | KY293076 | KY293126 |  |  |
|  |  |  |  |  | DC6 | ***KF021121*** |  |  |  |  |  |  |  |  |  |  |  |  |
|  |  |  |  |  | DC7 | ***KF021122*** |  |  |  |  |  |  |  |  |  |  |  |  |
|  |  |  |  |  | DC8 | ***KF021123*** | KY292674 | KY292727 | KY292777 | KY292826 | KY292877 | KY292923 |  | KY293022 | KY293077 | KY293127 | KY293166 |  |
|  |  |  |  |  | DC9 | ***KF021124*** |  |  |  |  |  |  |  |  |  |  |  |  |
| Hukou, Jiujiang, Jiangxi (26) | 29.7084 | 116.1846 | Yangtze R. | 6 | HK1 | ***KF021125*** |  |  |  |  |  |  |  |  |  |  |  |  |
|  |  |  |  |  | HK2 | ***KF021126*** |  |  |  |  |  |  |  |  |  |  |  |  |
|  |  |  |  |  | HK3 | ***KF021127*** |  |  |  |  |  |  |  |  |  |  |  |  |
|  |  |  |  |  | HK4 | ***KF021128*** |  |  |  |  |  |  |  |  |  |  |  |  |
|  |  |  |  |  | HK5 | ***KF021129*** | KY292693 | KY292737 | KY292786 | KY292837 | KY292883 | KY292934 | KY292981 | KY293035 | KY293087 | KY293134 |  |  |
|  |  |  |  |  | HK6 | ***KF021130*** |  |  |  |  |  |  |  |  |  |  |  |  |
| Yangtze, unknown (27) |  |  | Yangtze R. | 1 |  | **AY089714^2^** |  |  |  |  |  |  |  |  |  |  |  |  |
| Qiantangjiang, Zhejiang (28) |  |  | Qiantangjiang R. | 1 |  | **AY089715^2^** |  |  |  |  |  |  |  |  |  |  |  |  |
| Lingjiang, Zhejiang (29) |  |  | Lingjiang .R | 1 |  | **AY089716^2^** |  |  |  |  |  |  |  |  |  |  |  |  |
| Minjiang, Fujian (30) |  |  | Minjiang R. | 1 |  | **AY089717^2^** |  |  |  |  |  |  |  |  |  |  |  |  |
| Jiulongjiang, Fujiang (31) |  |  | Jiulongjiang R. | 1 |  | **AY089718^2^** |  |  |  |  |  |  |  |  |  |  |  |  |
| Out-group |  |  |  |  |  |  |  |  |  |  |  |  |  |  |  |  |  |  |
| *Hemiculter bleekeri* |  |  |  |  |  | **KF029693^3^** |  |  |  |  |  |  |  |  |  |  |  |  |
| *Hemiculter bleekeri* |  |  | Yangtze R. |  |  |  | KY292663 | KY292719 | KY292771 | KY292819 | KY292871 | KY292915 | KY292967 | KY293014 | KY293069 | KY293122 | KY293163 | KY293190 |
| *Culter alburnus* |  |  |  |  |  | **KM044500^4^** |  |  |  |  |  |  |  |  |  |  |  |  |

**Reference**

1. Fan Q & He S. (2014) The pattern of upper and middle yangtze drainages shapes the genetic structure and diversity of *hemiculter leucisculus* revealed by mitochondrial dna locus. *Acta Hydrobiologica Sinica*, 38(4):627-635.

2. Pi, B., Che, Y. F. & Zheng, G. C. Molecular evolution and biogeography of Hemiculter leucisculus (Teleostei: Cyprinidae) from South China, based on mitochondrial DNA information. Unpublished.

3. Tang, K.L., Agnew, M.K., Hirt, M.V., Lumbantobing, D.N., Sado, T., Teoh, V.H., Yang, L., Bart, H.L., Harris, P.M. & He, S. (2012) Limits and phylogenetic relationships of East Asian fishes in the subfamily Oxygastrinae (Teleostei: Cypriniformes: Cyprinidae). *Zootaxa*, **3681**, 101.

4. Chen, L. & Zhou, L. The complete mitochondrial genome of Culter alburnus (Cyprinidae: Cultrinae). Unpublished.
