# Supplementary material for: Phylogeographic structure, cryptic speciation and demographic history of the sharpbelly (Hemiculter leucisculus), a freshwater habitat generalist from southern China
Source: BMC Evol Biol. 2017 Sep 12;17:216. doi: 10.1186/s12862-017-1058-0 (PMC5596851; doi:10.1186/s12862-017-1058-0)
Supplement: Supplementary file 2 — Information of primer pairs used in our study. Table S3 Summary statistics for the 13 loci used in this study. bp, length of locus in bases; n, number of sequences; ps, polymorphic sites; pis, parsimony informative sites; LNR, length of the longest non-recombining regions, h, number of haplotypes. Table S4 Nucleotide substitution models used in tree reconstruction. Pinvar, proportion of invariable sites; Gamma, gamma shape parameter. Table S5 Nucleotide substitution models for Cytb and three nuDNA loci used in extend Bayesian skyline plots. Table S6 Genetic diversity statistics for each population based on Cytb. n, individual numbers; Nh, haplotype numbers; ph, private haplotype within each population; Hap, Cytb haplotype; h, haplotype diversity; π, nucleotide diversity. Table S7 Genetic distance based on Cytb among the three lineages estimated by K2P distance (%). Figure S1 The entire distribution of Hemiculter leucisculus in global scale. The map derived from http://www.discoverlife.org. (DOCX 267 kb) [file 12862_2017_1058_MOESM2_ESM.docx]

**Phylogeographic structure, cryptic speciation and demographic history of the sharpbelly (*Hemiculter leucisculus*), a freshwater habitat generalist from southern China**

Weitao Chen^1,2^, Zaixuan Zhong^1,2^, Wei Dai^1,2^, Qi Fan^1^ and Shunping He^1*^

^1^The Key Laboratory of Aquatic Biodiversity and Conservation of Chinese Academy of Sciences, Institute of Hydrobiology, Chinese Academy of Sciences, Wuhan, Hubei 430072, China

^2^University of Chinese Academy of Sciences, Beijing 100049, People’s Republic of China

**Table S2**: Information of primer pairs used in our study.

|  | Primer name | Primer sequence | Annealing  temperature (℃) | Cited sources |
| --- | --- | --- | --- | --- |
| Cytb | L14724 | GACTTGAAA AACCACCGTTG | 58-64 | Xiao *et al.* (2001)  Xiao *et al.* (2001) |
|  | H15915 | CTCCGATCTCCGGATTACAAGAC |  |  |
| EGR3 | E3 161F | AATATCATGGACYTGGGNATGG | 55 | Chen *et al.* (2008)  Chen *et al.* (2008) |
|  | E3 1136R | GGYTTCTTGTCCTTCTGTTTSAG |  |  |
| ENC1 | ENC1_F85 | GACATG CTGGAGTTTCAGGA | 53 | Li *et al.* (2007)  Li *et al.* (2007) |
|  | ENC1_R982 | ACTTGTTRGCMACTGGGTCAAA |  |  |
| Glyt | Glyt_F559 | GGACTGTCMAAGATGACCACMT | 55 | Chen *et al.* (2008)  Chen *et al.* (2008) |
|  | Glyt_R1562 | CCCAAGAGGTTCTTGTTRAAGAT |  |  |
| myh6 | myh6_F459 | CATMTTYTCCATCTCAGATAATGC | 53 | Chen *et al.* (2008) |
|  | myh6_R1325 | ATTCTCACCACCATCCAGTTGAA |  | Chen *et al.* (2008) |
| plagl2 | plagl2_F9 | CCACACACTCYCCACAGAA | 55 | Chen *et al.* (2008) |
|  | plagl2_R930 | TTCTCAAGCAGGTATGAGGTAGA |  | Chen *et al.* (2008) |
| Ptr | Ptr_F458 | AGAATGGATWACCAACACYTACG | 55 | Chen *et al.* (2008) |
|  | Ptr_R1248 | TAAGGCACAGGATTGAGATGCT |  | Chen *et al.* (2008) |
| RAG2 | RAG2-f2a | AARCGCTCMTGTCCMACTGG | 55 | Lovejoy & Collette (2001)  Lovejoy & Collette (2001) |
|  | RAG2-R6a | TGRTCCARGCAGAAGTACTTG |  |  |
| Rhodopsin | RH193F | CNTATGAATAYCCTCAGTACTACC | 55 | Chen *et al.* (2003) |
|  | RH 1039R | TGCTTGTTCATGCAGATGTAGA |  | Chen *et al.* (2003) |
| RYR3 | RYR3_F15 | GGAACTATYGGTAAGCARATGG | 55 | Chen *et al.* (2008) |
|  | RYR3_R968 | TGGAAGAAKCCAAAKATGATGC |  | Chen *et al.* (2008) |
| SH3PX3 | SH3PX3_F461 | GTATGGTSGGCAGGAACYTGAA | 55 | Chen *et al.* (2008)  Chen *et al.* (2008) |
|  | SH3PX3_R1303 | CAAACAKCTCYCCGATGTTCTC |  |  |
| sreb2 | sreb2_F10 | ATGGCGAACTAYAGCCATGC | 55 | Chen *et al.* (2008) |
|  | sreb2_R1094 | CTGGATTTTC TGCAGTASAGGAG |  | Chen *et al.* (2008) |
| zic1 | zic1_F9 | GGACGCAGGACCGCARTAYC | 57 | Chen *et al.* (2008) |
|  | zic1_R967 | CTGTGTGTGTCCTTTTGTGRATYTT |  | Chen *et al.* (2008) |

**Table S3**: Summary statistics for the 13 loci used in this study. bp, length of locus in bases; n, number of sequences; ps, polymorphic sites; pis, parsimony informative sites; LNR, length of the longest non-recombining regions, h, number of haplotypes.

|  | bp | n | ps | pis | LNR (bp) | h |
| --- | --- | --- | --- | --- | --- | --- |
| Cytb | 1040 | 390 | 213 | 150 | - | 171 |
| EGR3 | 883 | 55 | 27 | 24 | 632 | 10 |
| ENC1 | 795 | 51 | 18 | 14 | 659 | 17 |
| Glyt | 882 | 47 | 29 | 18 | 577 | 11 |
| myh6 | 752 | 51 | 40 | 28 | 429 | 11 |
| plagl2 | 698 | 43 | 39 | 24 | 485 | 11 |
| Ptr | 676 | 51 | 14 | 10 | 586 | 15 |
| RAG2 | 1227 | 46 | 73 | 57 | 450 | 13 |
| Rhodopsin | 743 | 54 | 26 | 22 | 522 | 14 |
| RYR3 | 830 | 52 | 25 | 19 | 543 | 13 |
| SH3PX3 | 704 | 40 | 29 | 15 | 618 | 15 |
| sreb2 | 951 | 26 | 15 | 9 | 951 | 12 |
| zic1 | 846 | 26 | 12 | 10 | 846 | 7 |

Table S4: Nucleotide substitution models used in tree reconstruction. Pinvar, proportion of invariable sites; Gamma, gamma shape parameter.

|  | | Best model | Pinvar | Gamma |
| --- | --- | --- | --- | --- |
| Cytb | GTR+I+G | 0.622 | 1.485 |  |
| EGR3 | GTR+I | 0.937 | - |  |
| ENC1 | GTR+I | 0.927 | - |  |
| Glyt | K80+I | 0.939 | - |  |
| myh6 | GTR+I+G | 0.842 | 0.688 |  |
| plagl2 | HKY+I | 0.894 | - |  |
| Ptr | HKY+I | 0.950 | - |  |
| RAG2 | GTR+I+G | 0.866 | 0.643 |  |
| Rhodopsin | HKY+I | 0.916 | - |  |
| RYR3 | HKY+I | 0.934 | - |  |
| SH3PX3 | HKY+I | 0.909 | - |  |
| sreb2 | HKY+I | 0.916 | - |  |
| zic1 | HKY | - | - |  |

**Table S5:** Nucleotide substitution models for Cytb and three nuDNA loci used in extended Bayesian skyline plots (EBSPs).

|  | Cytb | EGR | Ptr | RAG2 |
| --- | --- | --- | --- | --- |
| Lineage A | GTR+I | HKY | HKY | HKY |
| Lineage B | GTR+I | HKY | HKY | HKY+I |
| Lineage C | GTR+I+G | GTR+I | HKY+I | GTR+I+G |

**Table S6:** Genetic diversity statistics for each population based on Cytb. n, individual numbers; Nh, haplotype numbers; ph, private haplotype within each population; Hap, Cytb haplotype; *h*, haplotype diversity; π, nucleotide diversity.

| Locality | Nh/n | ph | Hap | Lineage | *h* | π |
| --- | --- | --- | --- | --- | --- | --- |
| 1 | 2/4 | 0 | 6;120 | C | - | - |
| 2 | 3/9 | 0 | 6;7;120 | C | 0.417±0.191 | 0.00085±0.00049 |
| 3 | 2/12 | 2 | 121;122 | C | 0.303±0.147 | 0.00117±0.00057 |
| 4 | 9/19 | 5 | 6;10;24;36-41 | C | 0.899±0.046 | 0.00752±0.00048 |
| 5 | 20/37 | 17 | 1-5;123;130-143 | B; C | 0.940±0.021 | 0.01411±0.00306 |
| 6 | 1/2 | 0 | 123 | B | - | - |
| 7 | 15/28 | 7 | 6-14;123-124;126-129 | B; C | 0.884±0.050 | 0.0235±0.00142 |
| 8 | 5/13 | 1 | 6;12;15;104;171 | A; C | 0.628±0.143 | 0.01386±0.00786 |
| 9 | 1/1 | 0 | 149 | A | - | - |
| 10 | 17/36 | 11 | 145;147;149;151-164 | A | 0.902±0.036 | 0.00752±0.00046 |
| 11 | 8/15 | 4 | 149;152-153;159;165-168 | A | 0.790±0.105 | 0.00723±0.00105 |
| 12 | 7/9 | 4 | 144-150 | A | 0.917±0.092 | 0.01047±0.00187 |
| 13 | 1/1 | 1 | 170 | A | - | - |
| 14 | 1/1 | 1 | 169 | A | - | - |
| 15 | 1/1 | 0 | 149 | A | - | - |
| 16 | 1/1 | 0 | 15 | C | - | - |
| 17 | 25/30 | 14 | 2;23;28;39;58;63;88;92-106 | C | 0.982±0.016 | 0.00665±0.00034 |
| 18 | 17/26 | 8 | 6-7;10-12;15;22;28;83-91 | C | 0.951±0.027 | 0.00638±0.00039 |
| 19 | 2/2 | 1 | 6;82 | C | - | - |
| 20 | 9/10 | 4 | 6;58;75-81 | C | 0.978±0.054 | 0.00729±0.00097 |
| 21 | 22/29 | 11 | 6;10;15-16;27;29;57;61;75;78;81;107-117 | C | 0.958±0.028 | 0.00646±0.00054 |
| 22 | 29/41 | 16 | 6;10;16;22;27-29;31;39;55-74 | C | 0.960±0.022 | 0.00976±0.00149 |
| 23 | 25/31 | 11 | 5-7;10;12;16-35 | C | 0.974±0.02 | 0.00587±0.0007 |
| 24 | 1/1 | 1 | 42 | C | - | - |
| 25 | 13/20 | 9 | 2;6;28-29;47-54;125 | C | 0.942±0.034 | 0.0105±0.00347 |
| 26 | 6/6 | 4 | 6;31;43-46 | B; C | 1.00±0.096 | 0.00609±0.00210 |
| 27 | 1/1 | 0 | 6 | C | - | - |
| 28 | 1/1 | 1 | 119 | C | - | - |
| 29 | 1/1 | 1 | 118 | C | - | - |
| 30 | 1/1 | 0 | 15 | C | - | - |
| 31 | 1/1 | 0 | 15 | C | - | - |
| Overall | 171/390 | 134 |  | - | 0.9737 ± 0.0043 | 0.0290 ± 0.0013 |

**Table S7**: Genetic distance based on Cytb among the three lineages estimated by K2P distance (%).

|  | A | B | C |
| --- | --- | --- | --- |
| A |  |  |  |
| B | 6.06±0.83 |  |  |
| C | 6.87±0.89 | 4.39±0.58 |  |

Figure S1: The entire distribution of *Hemiculter leucisculus* in global scale. The map derived from http://www.discoverlife.org.


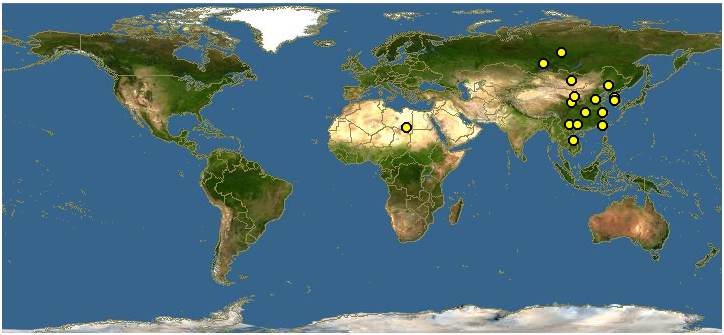


**Reference**

Chen, W. J., Bonillo, C. & Lecointre, G. (2003) Repeatability of clades as a criterion of reliability: a case study for molecular phylogeny of Acanthomorpha (Teleostei) with larger number of taxa. *Mol Phylogenet Evol*, **26**, 262-288.

Chen, W.J., Miya, M., Saitoh, K. & Mayden, R.L. (2008) Phylogenetic utility of two existing and four novel nuclear gene loci in reconstructing Tree of Life of ray-finned fishes: The order Cypriniformes (Ostariophysi) as a case study. *Gene*, **423**, 125-134.

Li, C.H., Orti, G., Zhang, G. & Lu, G.Q. (2007) A practical approach to phylogenomics: the phylogeny of ray-finned fish (Actinopterygii) as a case study. *BMC Evol Biol*, **7**

Lovejoy, N.R. & Collette, B.B. (2001) Phylogenetic relationships of new world needlefishes (Teleostei : Belonidae) and the biogeography of transitions between marine and freshwater habitats. *Copeia*, 324-338.

Xiao, W., Zhang, Y. & Liu, H. (2001) Molecular systematics of Xenocyprinae (teleostei: cyprinidae): taxonomy, biogeography, and coevolution of a special group restricted in East Asia. *Mol Phylogenet Evol*, **18**, 163-73.
